# Evaluating self-assistance during functional reach with a passive hydrostatic exoskeleton under artificial impairment

**DOI:** 10.1186/s12984-025-01696-8

**Published:** 2025-07-16

**Authors:** Julia Manczurowsky, Henry Mayne, David Nguyen, Meghan Kenney, John Peter Whitney, Christopher J. Hasson

**Affiliations:** 1 Department of Physical Therapy, Movement and Rehabilitation Sciences, Northeastern University, Boston, MA USA; 2 Department of Electrical and Computer Engineering, Northeastern University, Boston, MA USA; 3 Department of Mechanical and Industrial Engineering, Northeastern University, Boston, MA USA; 4 Institute for Experiential Robotics, Northeastern University, Boston, MA USA; 5 Department of Biological Sciences, University of Connecticut, Storrs, CT USA

## Abstract

**Background:**

Practicing functional upper-extremity tasks with manual self-assistance may promote motor recovery and restore voluntary control to an impaired limb, reducing reliance on external aid. However, most evidence comes from studies involving tasks with limited coordinative demands. In a functional task like reaching for and lifting an object, learning to generate coordinated assistive forces with an external device may pose bilateral sensorimotor challenges that limit motor learning in the impaired limb. To address this question, we developed a passive hydrostatic exoskeleton (hEXO) that enables self-assistance and paired it with an artificial impairment paradigm using Dysfunctional Electrical Stimulation (DFES), which induces involuntary hand closure during reaching.

**Methods:**

Twenty neurologically typical adults (26 ± 3 yrs) performed a reach-to-grasp and object lift task under challenging sensorimotor conditions: as fast as possible with their non-dominant hand while experiencing an artificial impairment induced by DFES. The stimulation functionally mimicked deficits related to a flexion synergy after neurological injury by making it difficult for participants to extend their fingers while reaching for an object. Experiment 1 assessed the short-term effects of DFES and wearing the hEXO. In Experiment 2, participants were randomly assigned to either a group that could self-assist with the hEXO (*n* = 10) or a control group that could not self-assist (*n* = 10) to investigate adaptation to self-assistance and transfer of motor performance to unassisted conditions.

**Results:**

DFES created a sensorimotor challenge and increased reach-to-grasp time by about 50% during early exposure. The self-assist group improved their reach-to-grasp times faster than controls (*p* = 0.008), achieved comparable reaching times (*p* = 0.060), and had a slightly higher incidence of unsuccessful attempts (about one in 20 attempts; *p* < 0.001). Reach-to-grasp performance did not decline following the removal of self-assistance, indicating no performance dependency. Both groups had similar movement times and success rates in the final unassisted practice block.

**Conclusions:**

In this sample of adults with an artificial impairment, self-assistance using a passive hydrostatic exoskeleton accelerated motor performance improvements without creating a dependency on the assistance. If replicated in clinical populations, this approach may help promote upper-limb functional independence.

**Supplementary Information:**

The online version contains supplementary material available at 10.1186/s12984-025-01696-8.

## Background

### Grasping impairments after stroke

Roughly 80% of stroke survivors have motor limitations in the upper limb that lead to slow, segmented, and indirect reaching movements [1] and a loss of prehensile control that impedes daily tasks [2] and reduces quality of life [3]. These deficits stem from a combination of reduced volitional finger extension [4, 5], often coupled with involuntary elbow, wrist, and finger flexion during arm extension [6]. Finger extension is more frequently affected than flexion after motor cortex lesions [7, 8], making it difficult for a person to open their hemiparetic hand and reach for an object [9], resulting in functional hand impairment [10]. Grasping deficits are often resistant to conventional rehabilitation [11], highlighting the need for treatments that explicitly consider sensorimotor feedback and corrective motor adjustments [1]. Interventions that target both grip formation and release [12] may improve functional outcomes [10], but a deeper understanding of the motor control strategies and learning mechanisms that support effective grip control is needed to guide such efforts.

Traditional rehabilitation approaches such as constraint-induced movement therapy, mirror therapy, and task-oriented training have been shown to drive structural changes in the brain and improve outcomes for retraining reach and grasp actions [13]. These strategies often include sensory stimulation, mental imagery, hand muscle strengthening, and pre-shaping the hand before grasping [14, 15]. Constraint-induced movement therapy [16] promotes use of the more-affected arm by restricting the less-affected one. Mirror therapy enhances sensory input and task-oriented training targets specific functional goals, each of which has been associated with improved performance in daily activities [3, 13, 17]. Although less common, therapy targeting the arm ipsilateral to the cortex lesion has been shown to improve functional independence in individuals with chronic stroke, without negatively affecting the contralateral, more impaired arm [13]. Such approaches suggest that training the ipsilateral hemisphere may help restore limb control and promote hemispheric synchronization [18, 19].

### Bimanual coordination and motor learning in rehabilitation

Both brain hemispheres contribute to hand movement, with the dominant hemisphere prioritizing control of movement trajectory, and the non-dominant hemisphere more involved with position control [18, 19]. During bimanual tasks, the limbs are not only physically connected but also coupled at sensorimotor and neuronal levels [20]. Stroke-related performance deficits are often present in both limbs [21], including increased muscle effort, altered recruitment patterns, and compensatory hyperactivity in the ipsilateral sensorimotor cortex, which recruits additional neurons including those for shoulder movements, to support impaired hand control [22]. As a result, bi-hemispheric reorganization expands the unaffected hemisphere’s role in controlling the more affected arm, with recovery largely depending on the stroke’s location and severity [7].

Given this bilateral involvement in movement control and the potential for interlimb interactions, bilateral training strategies may offer unique benefits. Symmetric bimanual movements can prime the motor cortex and supplementary motor area, potentially improving control of the hemiparetic arm through increased activation compared to unilateral movements [23–25]. Unlike unilateral training, bimanual practice also reduces intracortical inhibition [26], which is associated with improved kinematics and shorter movement times in the paretic limb [24]. Furthermore, using one hand to assist the other may help reorganize corticospinal pathways [27], allowing individuals to refine their motor control strategies. It is also possible that mechanically coupling the hands through exoskeletons may provide synchronized sensory feedback that may help in retraining the impaired limb [28]. Self-assistance could better align neural activity with natural movement patterns and could be more effective than external assistance from a therapist or robot [29]. Taken together, these findings support the value of neurorehabilitation approaches that train the hands together through multisensory, functional practice.

Motor adaptation, defined as the short-term process by which the nervous system updates internal models to reduce movement errors and optimize control, is a key mechanism underlying motor learning in rehabilitation [30, 31]. Self-assistance mediated by a passive exoskeleton may engage this process by mechanically coupling the impaired and unimpaired limbs, allowing the unimpaired side to provide both movement guidance and synchronized sensory feedback. These signals can serve as error information that drives internal model refinement [32], supported by neuroplastic changes that reinforce successful control strategies [33]. Theories such as stochastic optimal control offer a framework for understanding how consistent reach and grasp performance can be maintained despite natural motor variability [34]. Understanding these mechanisms of motor adaptation is essential for designing effective rehabilitation strategies. One approach to facilitating adaptation is through robotic systems, which can provide structured assistance while challenging the nervous system to refine motor control.

### Self-assisted rehabilitation

Robotic systems have shown promise in upper extremity rehabilitation by implementing key factors of effective movement recovery, including reducing neuromotor demands, enabling earlier and more intense interventions, and allowing enriched whole-task practice of real-world behaviors that would otherwise be unattainable without robotic assistance [35]. This includes robot-mediated bimanual symmetric tasks, which emphasize simultaneous bilateral training where the unaffected hand guides the movement of the affected hand [36]. However, excessive robotic support can lead to “slacking,” where reduced muscle activity and engagement hinder motor adaptation [37, 38]. A passive, unpowered exoskeleton allowing self-assistance, i.e., using the less-affected limb to assist the more-affected one, may reduce slacking by requiring active effort for assistance. Previous work with a bilateral cable-driven passive exoskeleton showed less motor slacking during self-assisted elbow motion compared to externally powered unilateral practice [38]. However, the effects of self-assisted bimanual motion in more complex functional tasks remain unclear. Tasks like reaching and grasping have higher spatiotemporal demands, making accurate haptic feedback essential. When self-assistance requires coordinating both hands, it could introduce a “learning cost,” where users underperform due to the added complexity of bimanual control. The key question is whether the benefits of self-assistance outweigh the learning costs in functional tasks.

To address this question, we built a hydrostatic exoskeleton (hEXO) that transfers forces between a user’s hands using water and air, allowing one hand to manually assist the other in grasping objects. The exoskeleton is passive and contains no electronics or motors. Hydrostatic force transmission is well-suited for self-assisting grasping due to its high transparency and haptic fidelity. We used the hEXO in a pilot study with healthy participants to investigate how individuals adapt to self-assistance during a goal-directed reach-to-grasp and object lift task. To simulate impaired motor function, we introduced a temporary and controlled impairment by applying Dysfunctional Electrical Stimulation (DFES) to participants’ left hand. DFES caused involuntary hand closure, disrupting the normal sequence of opening the hand during forward reach. This model system provides a controlled platform for examining how self-assistive strategies develop and adapt. A foundational understanding of self-assistance strategies is essential before running trials with clinical populations, where impairments are heterogeneous and often less predictable.

### Experimental aims

In one experiment, we investigated how DFES and wearing the hEXO (without self-assisting) affected performance in the reach-to-grasp and object lift task, both independently and in combination. In a second experiment, we tested whether self-assistance would accelerate performance improvements through bimanual facilitation, which we define as enhanced coordination or neural priming resulting from the active use of both hands, including the transmission of force and sensory information from the unimpaired to the impaired limb via the hEXO. The alternative hypothesis is that the added challenge of coordinating both hands via the exoskeleton would negate bimanual facilitation benefits. We examined how participants adapted to self-assistance by examining changes in movement times, success rates, hand shaping, and muscular activity. To test if self-assistance is vulnerable to motor slacking, we assessed whether participants developed a dependency on self-assistance by evaluating their performance after its removal following a period of self-assisted practice (i.e., unassisted transfer).

## Methods (Apparatus)

### The passive hydrostatic exoskeleton

**Fig. 1:**
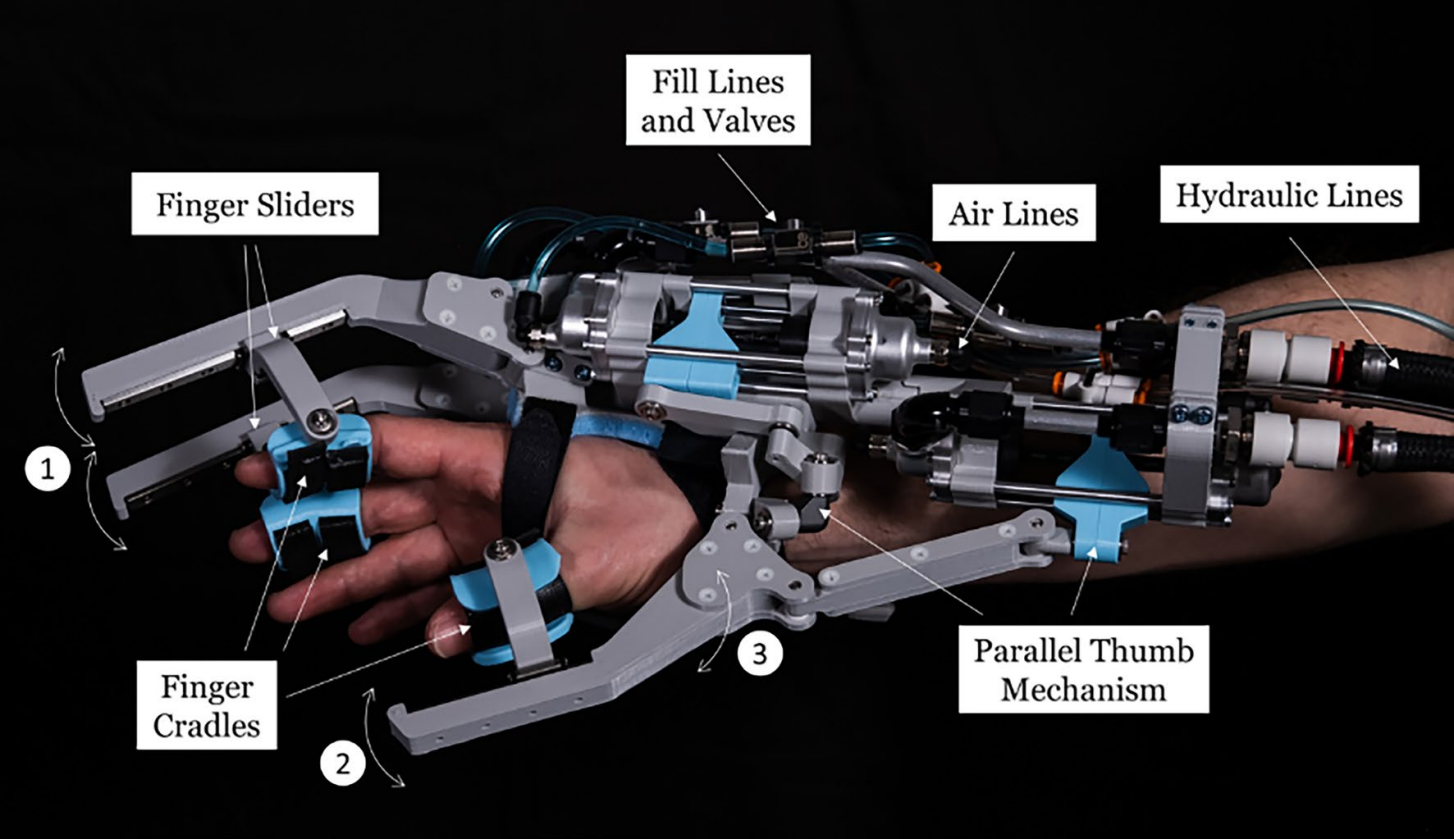
One hydrostatic exoskeleton (hEXO) glove of a paired bimanual system. The air and hydraulic lines lead to another, mirrored hEXO glove worn on the other hand (not shown). (1) The first and second finger flexion/extension are coupled. (2) The thumb can independently flex/extend, and (3) abduct/adduct. See text for more details on the device design and operation

To demonstrate proof-of-concept of a passive hydrostatic exoskeleton (hEXO) for self-assisted rehabilitation, we adapted an existing system used in the ANA Avatar telepresence robot competition, AVATAR 2 [39–41]. Each exoskeleton glove has three degrees of freedom (DoFs), two for the thumb and one synchronizing the index and middle fingers (Fig. 1). These paired gloves are equipped with a hydrostatic transmission to provide high-fidelity force feedback between each pair of actuated fingers and thumb. Using this transmission, a driving hand can transfer forces to an impaired hand without the need for motors, external power sources, or electronics.

#### Hydrostatic transmission

**Fig. 2:**
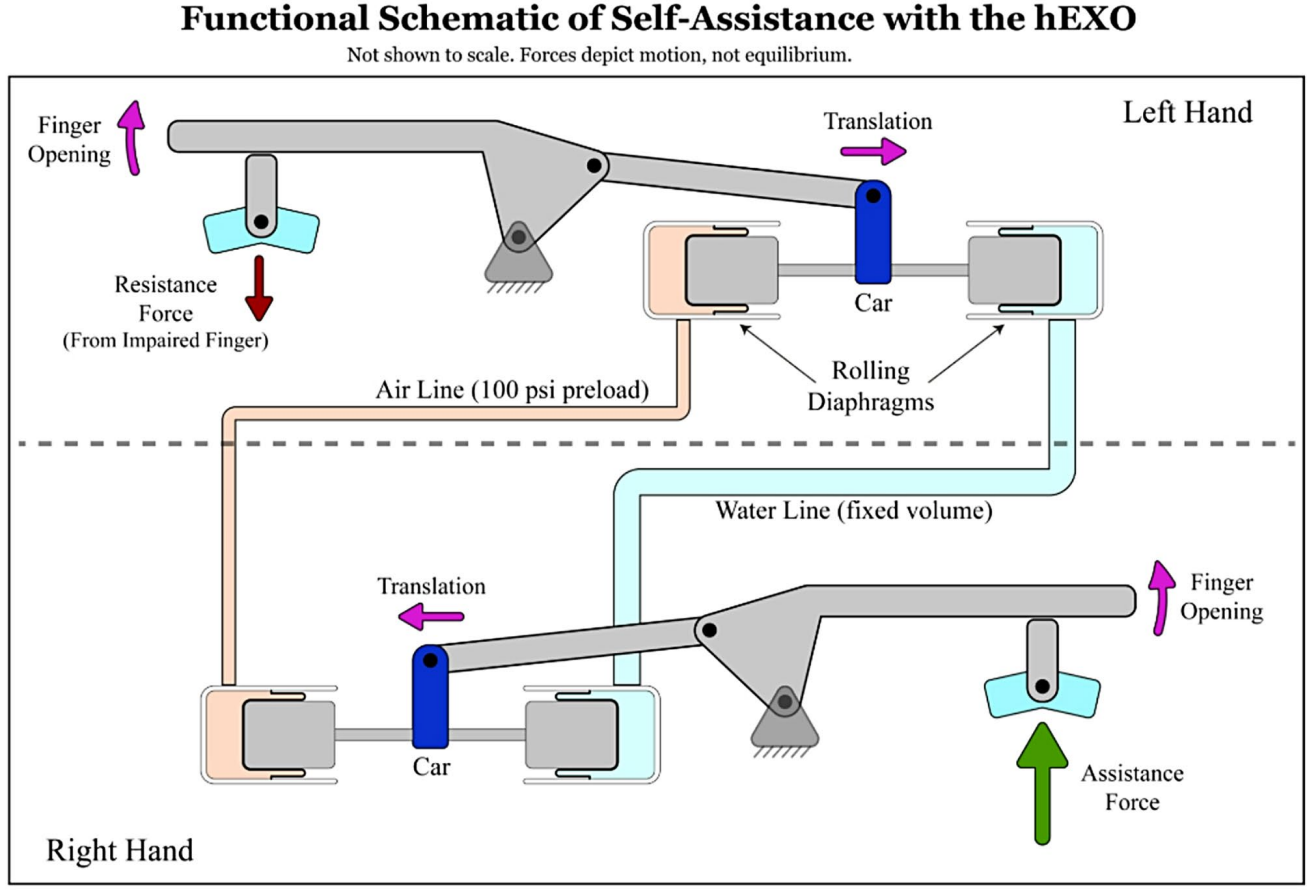
Functional schematic of transmission in a single degree of freedom of the hydrostatic exoskeleton (hEXO) glove system. This diagram illustrates how volitional movement of one hand (here, right) provides assistance to the opposite hand (here, left) through a hydrostatic transmission. A pneumatic line (100 psi preload) maintains system pressure, while a sealed hydraulic line enables displacement coupling between linked mechanical “cars” (dark blue). In this demonstrative example, an unbalanced force at the finger is shown to illustrate when the unimpaired finger generates more force than the impaired finger’s resistance, the result is finger opening and transmission of motion across the system. Note that this schematic is intended to show general hEXO operational features and therefore does not show internal forces acting on the system and other details

The hydrostatic transmission is a hybrid air-water system that connects the gloves together using two paired actuators for each degree of freedom (DoF; Fig. 2). Each actuator features a pair of rolling diaphragm pistons, with a fixed-volume water line on one side and an air line on the other. The air line provides a 100-psi preload. The pair of pistons is attached to a “car” (depicted in dark blue in Fig. 2), which rides on a set of linear rails to create a prismatic joint with a stroke of 24 mm. In the present configuration, when the right hand assists the left hand in closing (grasping), the water line pressure locally increases; when the right hand assists the left hand in opening, the water line pressure locally decreases. Due to hose nonlinear stiffness properties or latent air bubbles in the water line, in a right-hand assisting the left situation, grasping assistance may have a higher coupling stiffness than hand opening assistance. The actuators weigh 95 g each (including the support structure) with a 400:1 bidirectional strength-to-weight ratio (+/- 230 N). Their advantages include the same high stiffness of traditional hydraulic actuators with better sealing, less friction, and low operating pressures. This enables each DoF to transmit high-frequency force feedback between the hands. Actuators like these have been used in teleoperated robotics [42–45]. The exact actuators used here were used in the existing AVATAR 2 system [39–41].

#### Exoskeleton glove integration

Each exoskeleton glove was designed with three DoFs, with one hydrostatic actuator for each DoF (six actuators for the bimanual system). The index and middle fingers are coupled with a single DoF, moving together in flexion/extension (Fig. 1; #1). The thumb has two DoFs: one for abduction/adduction (Fig. 1; #2) and one for flexion/extension (Fig. 1; #3). Each hydrostatic actuator is connected to the respective actuator on the other glove through aramid-wrapped rubber tubes for both air and water lines. A user’s hand is positioned between a memory foam layer on the back of the hand and a plastic palm piece with a set of hook-and-loop straps (Fig. 1). Crossing elastic drawstrings allow the palm piece to be tightened for a secure fit. Each actuated fingertip is fastened into cradles using softer hook-and-loop straps. The finger cradles provide support and attach each finger to the glove, which allows the finger to actuate the exoskeleton. The cradles translate along tracks, called finger sliders, as the user opens and closes their hand, enabling natural finger trajectories, accommodating a range of hand sizes, and improving the hEXO’s comfort and transparency. For the hEXO thumb’s two DoF, both actuators were grounded to the dorsum of the hand, and a parallel mechanism was designed that uses a spherical-four-bar mechanism for thumb abduction/adduction in line with a three-bar mechanism for thumb flexion/extension.

## Methods (General experimental)

### Study design and participants

A total of 20 neurologically typical adults (26 ± 3 yrs., 170.5 ± 8.9 cm tall, 4 female/16 male) participated in the study. Two related experiments were conducted, in which participants were asked to complete a reach-to-grasp and object lift task. To increase task difficulty, they used their non-dominant hand. Additionally, a temporary artificial impairment was applied to the non-dominant hand, causing it to close during the reach phase. This impairment was intended to further challenge performance and allow room to observe improvements over time.

In Experiment 1, all 20 participants performed the same experimental conditions to test the short-term effects of the artificial impairment and wearing the hEXO on the left hand (without self-assistance), both independently and in combination. Experiment 2 was designed to test the hypotheses about adaptation to self-assistance. For Experiment 2, the same 20 participants were randomly assigned to either a self-assist group (*n* = 10) or a control group (*n* = 10). The self-assist group was instructed to assist themselves using the coupled hand exoskeleton, and the control group wore the exoskeleton on the left hand only so they could not self-assist. All participants were right-handed, as determined by their self-reported writing hand. We chose to recruit only right-handed individuals to control for the small asymmetries in the exoskeleton’s mechanical transmission, where the stiffness may differ slightly depending on which hand is driving the system (see Methods/Apparatus).

Participants were screened for exclusion criteria, including a history of vasovagal responses, closed (e.g., rash) or open (e.g., wound) lesions of the skin over areas involved in the protocol, impaired sensation to light touch (e.g., neuropathy, carpal tunnel syndrome, diabetes), current pain (acute or chronic), implanted electrical devices (e.g., pacemaker, pain pump), allergy to disposable electrode or tape adhesive, or any hand deformity and/or previous arm/hand surgery. All study procedures were approved by the local institutional review board, and all participants provided informed consent. One participant was recruited and consented but did not perform the experimental protocol due to technical issues with the experimental setup.

### Experimental setup

#### Artificial impairment

We gave participants a temporary artificial impairment in their left hand, using what we term Dysfunctional Electrical Stimulation (DFES). This approach contrasts with Functional Electrical Stimulation (FES), which aims to restore, rather than disrupt, motor function [46]. We previously used DFES to impair walking [47]. In this study, we adapted DFES to impair the reach-to-grasp portion of the task by involuntarily activating the wrist and hand flexor muscles, thereby causing the hand to close during reaching. Thus, participants needed to activate their wrist and hand extensors to prevent the hand from closing before grasping the object.

Our aim with DFES was to functionally mimic deficits related to a flexion synergy common after neurological injury, including disrupted volitional reach-to-grasp sequencing. This opposition between intended extension and imposed closure of the hand creates a functional constraint akin to a synergy-driven impairment, where hand closure interferes with coordinated reach and grasp execution because of involuntary flexion force at the wrist and fingers [4]. DFES is not intended to replicate the full spectrum of sensorimotor deficits typically seen in a hemiparetic arm after neurological injury, including shoulder and elbow movement fractionation [6].

To employ DFES, two electrodes were used to stimulate the flexor muscles of the left index, middle finger, and thumb. The proximal electrode (size = 2.5 inches square) was placed half an inch below the elbow on the ventral side of the left forearm. The distal electrode (1 inch square) was placed on the thenar eminence of the left-hand lateral to the hand’s midline and distal to the wrist. Electrodes were attached to the stimulator via wire, with the positive terminal attached proximally and the negative terminal distally. After securing the electrodes, participants were assisted in donning gloves with rubberized fingertips, which they wore for the entire protocol. This also facilitated a snug fit of the hEXO gloves during the conditions when the device was worn. A volar wrist splint was fitted on the left hand to limit wrist flexion and extension during trials without the hEXO, so the action of DFES would be focused on the hand, rather than the wrist (the splint also mimicked the wrist-stabilizing effects of the hEXO when the hEXO was worn). DFES was delivered using a linear isolated stimulator (STIMISOLA, Biopac, Goleta, CA), which was controlled by an analog voltage provided by a function generator (Model 33210 A, Agilent, Santa Clara, CA).

The stimulation waveform consisted of a 2500 Hz bi-phasic sinusoidal carrier waveform, which delivered current in bursts of 25 cycles at a frequency of 50 bursts/second (period = 20 ms). The function generator was controlled by a custom MATLAB program through a GPIB (General Purpose Interface Bus). For stimulation calibration, participants were asked to relax while the current amplitude was increased in a stepwise fashion starting at 10 mA with 2 mA increments until the hand passively closed, i.e., the left thumb involuntarily contacted either the first or second finger. Once hand closure was achieved, we measured the stimulation-produced grasping force with a six-axis load cell (SI-65-5; ATI, Inc.). We continued to increase the stimulation current in 2 mA increments until a minimum passive grasping force of 6 N was achieved. We chose 6 N as it is close to the average grip force for grasping a similarly weighted/textured object to ours, as reported by Johansson and Westling [48]. For all remaining trials in the protocol where DFES was provided, we used the lowest possible current that elicited a response during calibration that met the perturbation criteria.

#### Electromyography (EMG)

For Experiment 2, the right forearm muscle activity was measured to assess the relative degree of assistance participants provided to their left hand through the hEXO transmission. Skin preparation involved the use of an exfoliating paste, followed by skin cleaning with alcohol. Two pairs of circular, pre-gelled, foam/adhesive-backed, latex-free, disposable Ag/AgCl snap electrodes (1 cm diameter; H124SG, Medtronic, Minneapolis, MN) were placed with an interelectrode spacing of 1 cm. Guided by Zipp [49] and using palpation against resisted isometric muscle contractions, one pair of electrodes was placed on the right extrinsic finger extensors, targeting the belly of the extensor digitorum communis, and a second pair was placed over the right extrinsic finger flexors, targeting the belly of the flexor digitorum superficialis.

EMG calibration was completed by having participants perform functional isometric maximal voluntary contractions (MVCs), so their right arm EMG could be expressed as a percentage of each participant’s functional MVC. Two hand flexor MVCs were performed first by having the participant grasp and squeeze a wooden cylinder as hard as possible for 2 s, with a 10 s rest between MVCs. Next, two hand extensor MVCs were performed. For these trials, the participant kept grasping the object, and the experimenter placed their hands over the participant’s hand. The participant then attempted to open their hand as hard as possible for 2 s against the resistance, again with a 10-second rest between maximum voluntary contractions (MVCs). We selected the maximum flexor and extensor MVC values from the four MVC attempts to normalize EMG during self-assistance in Experiment 2. The EMG data were collected using a wireless EMG system (Myon 320, Baar, Switzerland; bandwidth: 5–1,000 Hz, latency: 16 ms) at a rate of 1000 Hz.

#### Hydrostatic exoskeleton (hEXO)

For conditions in which participants donned the hEXO, the experimenter assisted with donning. The air and water tubes were run behind the participants’ backs and supported by a hook suspended from the ceiling. The weight of each hEXO glove was offloaded using 50 ft long elastic tubing (THERABAND tubing; red color; Akron, OH) suspended from the ceiling using a pulley system. The tubing length was chosen because having a very long cable provides a stable offloading force, such that any changes in the tubing length would produce very small changes in the offload force. Prior to each participant beginning the experiment, the thumb and finger positions on the hEXO gloves were synchronized using the water fill lines and valves on each glove to balance the amount of water between each actuator pair and produce symmetrical motion. In addition, the air pressure was “topped up” to the 100-psi preload.

#### Functional task

**Fig. 3:**
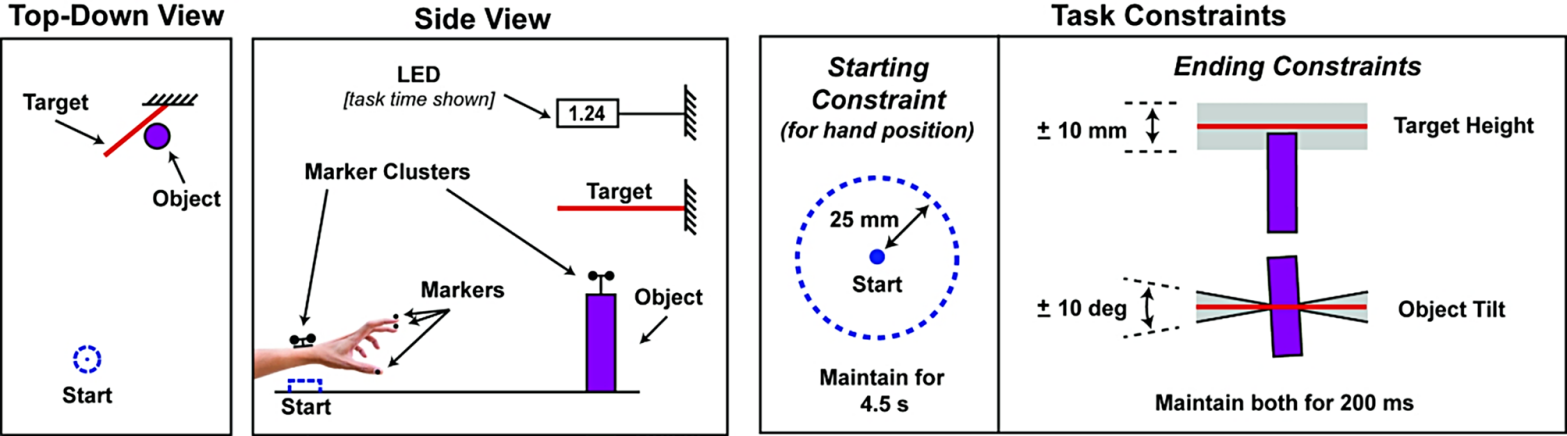
Schematics of the task layout and constraints. Passive reflective motion capture markers on the hand, fingers, object, and target tracked movements to measure task performance. In some conditions, participants wore a hydrostatic exoskeleton (hEXO) on one or both hands (not shown)

We used a functional reach-to-grasp and object lift action to emulate the types of tasks frequently encountered during everyday life. This type of task has been commonly used to study bimanual motor adaptation in healthy individuals and patient populations [50]. The task occurred in three dimensions using a physical object (Fig. 3). Participants sat in a chair with an erect posture, placing both of their arms on the table and their torsos positioned close to the table’s edge. The task required participants to reach forward with their left (non-dominant) hand, grasp a cylindrical object (diameter = 5.1 cm; length = 15.5 cm; mass = 0.220 kg), and lift the object to a target height (25 ± 1 cm) while keeping the object level (0 ± 10° of tilt in the horizontal plane). The height and tilt requirements had to be satisfied for 200 ms to successfully end the trial. The object distance was 31 cm from the starting position, which was well within arm’s reach for all participants. Participants viewed a small LED display positioned at eye level, which displayed the total completion time for the reach-to-grasp and object lift task.

To make the task challenging, participants were instructed to perform the task and achieve the goal as quickly as possible. Our pilot work showed that 2.0 s was short enough to constrain the number of potential successful movement strategies, yet long enough to prevent participants from becoming frustrated by a disproportionate number of unsuccessful attempts. Therefore, the maximum time for participants to complete the attempt was set to 2.0 s. Otherwise, the trial timed out and was flagged as an unsuccessful task. This time limit also set an upper bound for the duration of participants’ exposure to DFES if the participant lost their grip or fumbled with the object.

**Fig. 4:**
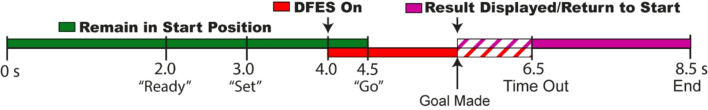
Timeline of a single reach-to-grasp and object lift attempt. Participants held their hand in the start position for 4.5 s (solid green). Dysfunctional Electrical Stimulation (DFES) was applied after 4.0 s and remained on during the attempt (solid red), ending either when the goal was reached or the trial timed out at 6.5 s (hatched red). They initiated the reach-to-grasp and object lift attempt as fast as possible on the “Go” command at 4.5 s. Feedback of task time was displayed immediately after success (hatched purple) or after timeout (solid purple), with all trials ending at 8.5 s. During feedback, participants returned to the start position

A timeline of the events for a single reach-to-grasp and object lift attempt is shown in Fig. 4. Before starting each attempt, participants placed their left hand in the starting position. Their arm had to remain within a 25 mm radius of the center of the starting circle for 4.5 s until a “go” cue. If they moved out of the start position before this time, the timer automatically paused, and the timer resumed when they reentered the starting position. After 2.0 s had elapsed in the starting position, they heard a “ready” command, followed by a “set” command at 3.0 s. At 4.0 s, in DFES conditions, the DFES turned on. Then, at 4.5 s, the “go” command was given, after which the participants had 2.0 s to complete the reach-grasp and object lift task. The DFES turned on before the “go” command to account for the time it takes for muscles to become activated upon being excited by DFES. When the goal criteria were satisfied or the attempt timed out, whichever came first, the DFES turned off, and the movement time was displayed on the LED. If the attempt timed out, i.e., the goal criteria were not satisfied in 2.0 s, a buzzer sounded, and “tout” (for “timed out”) was displayed. If the goal was achieved a bell sounded. After the goal was achieved or timed out, the participant moved back to the starting position. Once a total of 8.5 s had elapsed from the start of the attempt and the participant returned to the start position, the timer reset, and the next attempt began.

#### Motion capture

Control of the task, gating of the artificial impairment, and display of participant feedback was done by a custom MATLAB program at 60 Hz. This included processing streamed 6 DoF motion of the left arm and object, which was tracked online using Motive (v2.3.4, NaturalPoint, Inc., Corvallis, OR). Real-time optical motion capture was used to track hand and object movement and monitor adherence to task constraints, e.g., achieving the target ending position and returning to the correct starting position. Clusters of passive reflective markers were used to track the 6 DoF motion of the left forearm and the object. In attempts where the participants did not wear the hEXO, the forearm cluster was placed on the wrist splint above the radial styloid process. When wearing the hEXO, a set of four fixed markers on the exoskeleton over the forearm was tracked, as the device obscures the forearm. For the object, the cluster was mounted on a rigid post raised 4 cm above the top surface of the object. A single passive reflective marker was placed over the fingernail on the thumb, middle, and index fingers of each hand; these digits were of prime interest as they actuated the hEXO when self-assisting.

## Methods (Experiment 1)

### Protocol

This experiment was designed to test the short-term effects of DFES and wearing the hEXO (without self-assistance) on the performance of the reach-to-grasp and lift task. In other words, to determine how the DFES perturbation and the hEXO’s added inertia and mechanical constraints affect task performance. Quantifying this impairment also provided a baseline performance level for each participant, which is used in Experiment 2. All 20 participants first completed a familiarization block of 20 reach-to-grasp and object lift attempts without any experimental interventions. Next, participants performed one block of practice for four conditions (20 attempts each) in a randomized order:

- ***CTRL****(Control)*: Same as familiarization, i.e., the task is performed without wearing the hEXO and without DFES.
- ***DFES Only***: Participants performed the task while receiving DFES, which created an involuntary closure of the participant’s left hand. This assessed the effects of DFES on task performance.
- ***hEXO Only***: Participants performed the task while wearing the hEXO on the left hand only. This assessed the effects of wearing the hEXO on task performance (e.g., inertial and motion restrictions), in the absence of assistance forces.
- ***DFES + hEXO***: Participants performed the task while wearing the hEXO on the left hand while also receiving DFES on the left hand. This assessed the interactive effects of wearing the hEXO and receiving DFES on task performance.

### Data reduction

#### Motor performance

Overall task performance was quantified by the total reach-to-grasp and object lift task completion time, *TaskTime*, which was directly logged by the data collection program. Participants saw this time at the end of each trial on the visual display. This time was calculated as the time from when the hand broke through the 25 mm radius starting circle to when the task constraints were met (including object height and tilt requirements).

Because the artificial impairment caused the hand to involuntarily close when attempting to grasp the object, the reach-to-grasp phase of the movement was of particular interest. Therefore, we quantified the reach-to-grasp time, *ReachTime*. This time was calculated as the interval from movement onset to when the object was lifted 2 cm off the table, signifying a successful grasp. For *ReachTime*, we took advantage of the ability to perform an off-line analysis to have an earlier detection of movement onset (compared to *TaskTime*, which used the 25 mm radius breakthrough because of the constraints of online movement detection). *ReachTime* onset was defined as the start of the forward hand movement.

Using the measured forearm motion, derived from the displacement of the marker cluster placed on the forearm or on the hEXO when worn, we identified this starting point by backtracking from the time of peak hand velocity and stopping when the velocity crossed zero. A similar back-tracking approach was used by Mazzoni et al. [51], who used a 0.02 m/s velocity threshold, as they employed low-friction air-sleds to support the arm (the higher, non-zero threshold helps account for small variations in arm position due to the very low-friction environment). In our study, participants’ arms were resting on the table prior to movement, so their hand velocity was reliably zero before the reaching action - or more accurately, oscillated near-zero according to the spatial and temporal resolutions of the motion capture system, which were ± 0.0002 m (worst-case; specified by the manufacture) and 0.0083 s (120 Hz sampling rate), respectively, giving a resting “velocity noise” of about ± 0.00017 m/s. The reach-to-grasp termination was set as the first sample at which the object’s vertical height exceeded 2 cm above the table.

To understand how the experimental conditions affected the likelihood of overall task success, the percentage of successful reach-to-grasp and lift attempts, *PcntTaskSuc*, was calculated. This measure included all attempts in which the object was lifted to the target height (25 ± 1 cm), within the tilt tolerance (0 ± 10°), and within the 2.0 s time limit. We also calculated the percentage of successful reach-to-grasp actions, *PcntReachSuc*, which included attempts that may not have achieved the overall goal (i.e., did not satisfy the object height and tilt requirements and time limit), but achieved a successful grasp (lifted the object at least 2 cm).

#### Hand aperture

Hand aperture assessed how participants adjusted a key kinematic feature of the task: opening the hand to grasp the object against the artificial impairment that involuntarily closed the hand. We defined hand aperture as the magnitude of a three-dimensional vector from the thumb tip marker to a virtual point midway between the markers on the tips of the index and middle fingers. For these measures, gaps in the individual marker trajectories were filled in MATLAB using a piecewise cubic spline interpolation. After gap filling, the finger displacement data were filtered with a zero-lag low-pass 4th-order Butterworth filter with a 6 Hz cutoff frequency. For each reach, the initial aperture at movement onset, *InitAper*, and the peak aperture during the reach, *PeakAper*, were calculated. As with *ReachTime*, the aperture variables included all attempts where a grasp was achieved, regardless of whether the overall task was successful.

### Statistics

Within each participant, the values of the time and motion variables, *TaskTime*, *ReachTime*, *InitAper*, and *PeakAper*, were averaged across each block of 20 reach-to-grasp-and-lift actions. To assess whether these block-level averages were normally distributed, we used the Lilliefors test, an adaptation of the Kolmogorov-Smirnov test that accounts for unknown population parameters. All variables were normally distributed (*p* > 0.05). Outlier participants were identified using the interquartile range (IQR) method. Specifically, participant data points more than 1.5 × IQR above the third quartile or below the first quartile were considered outliers. For *TaskTime*, two outlier participants were identified in the CTRL condition, one in the DFES condition, and one in the DFES + hEXO condition. For *ReachTime*, one outlier participant was identified in the CTRL condition. For *InitAper*, two outlier participants were identified in the hEXO condition and one in the CTRL condition, whereas one outlier was identified for *PeakAper* in the DFES condition. These outliers were excluded from statistical analyses.

Statistical analyses were conducted using generalized linear mixed-effects models (GLMMs) in SPSS (v25, IBM, Armonk, NY; RRID: SCR_002865). The models included two within-participant factors with repeated measures: DFES (on/off) and hEXO (on/off). Separate linear models were run for each dependent measure: *TaskTime*, *ReachTime*,* InitAper*, and *PeakAper*. A heterogeneous compound symmetry covariance structure was used whenever it resulted in a lower Akaike Information Criterion (AIC) and Bayesian Information Criterion (BIC); otherwise, a diagonal covariance structure was used. For all models, multiple pairwise comparisons were adjusted using the sequential Bonferroni correction [52]. These adjusted *p*-values are reported.

We assessed the normality of the percentage-based measures (*PcntTaskSuc* and *PcntReachSuc*) using the Shapiro-Wilk test due to the bounded nature of the data (0-100%). In all conditions, the Shapiro-Wilk test indicated non-normality (*p* < 0.05) with an average skewness of 1.37 and 1.86 for *PcntTaskSuc* and *PcntReachSuc*, respectively. We log-transformed this data and identified outliers using a higher threshold of 3 × IQR, due to the boundedness and skewness. Due to being classified as outliers, one participant was excluded for *PcntTaskSuc* in the DFES + hEXO condition, and two participants were excluded for *PcntReachSuc* in the CTRL condition. A programming error caused one participant’s reach-to-grasp attempts in the DFES + hEXO to be signaled as unsuccessful. Because the task success was not computed correctly for this block, this participant’s *PcntTaskSuc* was excluded from the analysis. The issue was identified during data collection and corrected immediately after the block.

For the *PcntTaskSuc* and *PcntReachSuc* statistical analyses, a similar model structure was used as for *TaskTime*, *ReachTime*,* InitAper*, and *PeakAper;* however, a gamma distribution with a log link function was used to account for the skewed distributions. Repeated measures were initially included in the *PcntTaskSuc* and *PcntReachSuc* models but were removed when they did not improve AIC/BIC and led to convergence issues. Sequential Bonferroni corrections were performed, and adjusted *p*-values are reported.

## Methods (Experiment 2)

**Fig. 5:**
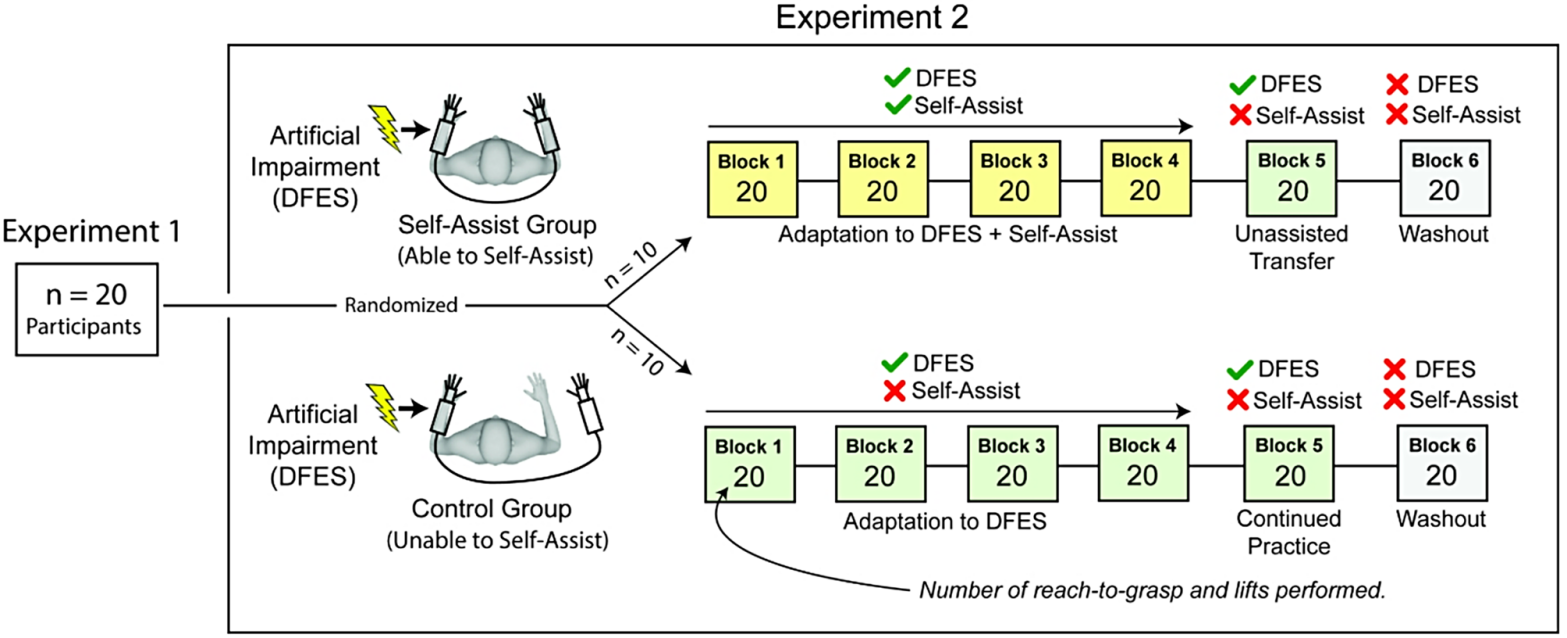
Experiment 2 protocol. The artificial impairment was induced by Dysfunctional Electrical Stimulation (DFES). The self-assist group wore the hydrostatic exoskeleton (hEXO) on both hands and were instructed to use their unimpaired right hand to assist their artificially impaired left hand while practicing the task. The control group did not wear the hEXO on their unimpaired hand and therefore could not self-assist

Our second experiment tested the hypothesis that participants who could self-assist during a reach-to-grasp and lift task would show faster performance improvements than a control group who could not self-assist. This experiment involved the same 20 participants who completed Experiment 1, who were then randomly assigned to either the self-assist or control groups. The self-assist group wore the hEXO on both hands and were instructed to use their right hand to assist their left hand in performing the task (Fig. 5). No explicit instructions were given regarding assistance techniques. The control group practiced the task while wearing the hEXO on the left hand but not the right hand and were therefore unable to assist themselves. This was done because there was no way to decouple the hEXO gloves. Both groups were instructed to keep their right forearm on the table, so they did not reach with their right hand. This also avoided the confound of the self-assist group contending with the added inertia of the hEXO on the right arm, while the control group did not, which would occur if participants mirrored the reaching motion of the left arm.

After donning the hEXO per their group assignment, both groups performed four practice blocks (Blocks 1–4), with 20 reach-to-grasp-and-lift attempts in each block, while being artificially impaired by DFES (Fig. 5). After completing the four practice blocks, participants in the self-assist group removed the hEXO from the right hand so they were no longer able to self-assist. Both groups then performed 20 attempts in the fifth practice block (Block 5) wearing the hEXO only on the left hand and receiving DFES. In a final washout block (Block 6), all participants removed the hEXO completely and practiced the task without DFES, so they could return to their normal, unperturbed task performance. For Experiment 2, all dependent variables were normalized to each participant’s performance in the DFES + hEXO condition (in Experiment 1) to control for individual differences in skill, the effects of the DFES, and the effects of wearing the hEXO (thus, we refer to these measures as “relative” to denote this normalization).

### Data reduction

#### Motor adaptation rate

**Fig. 6:**
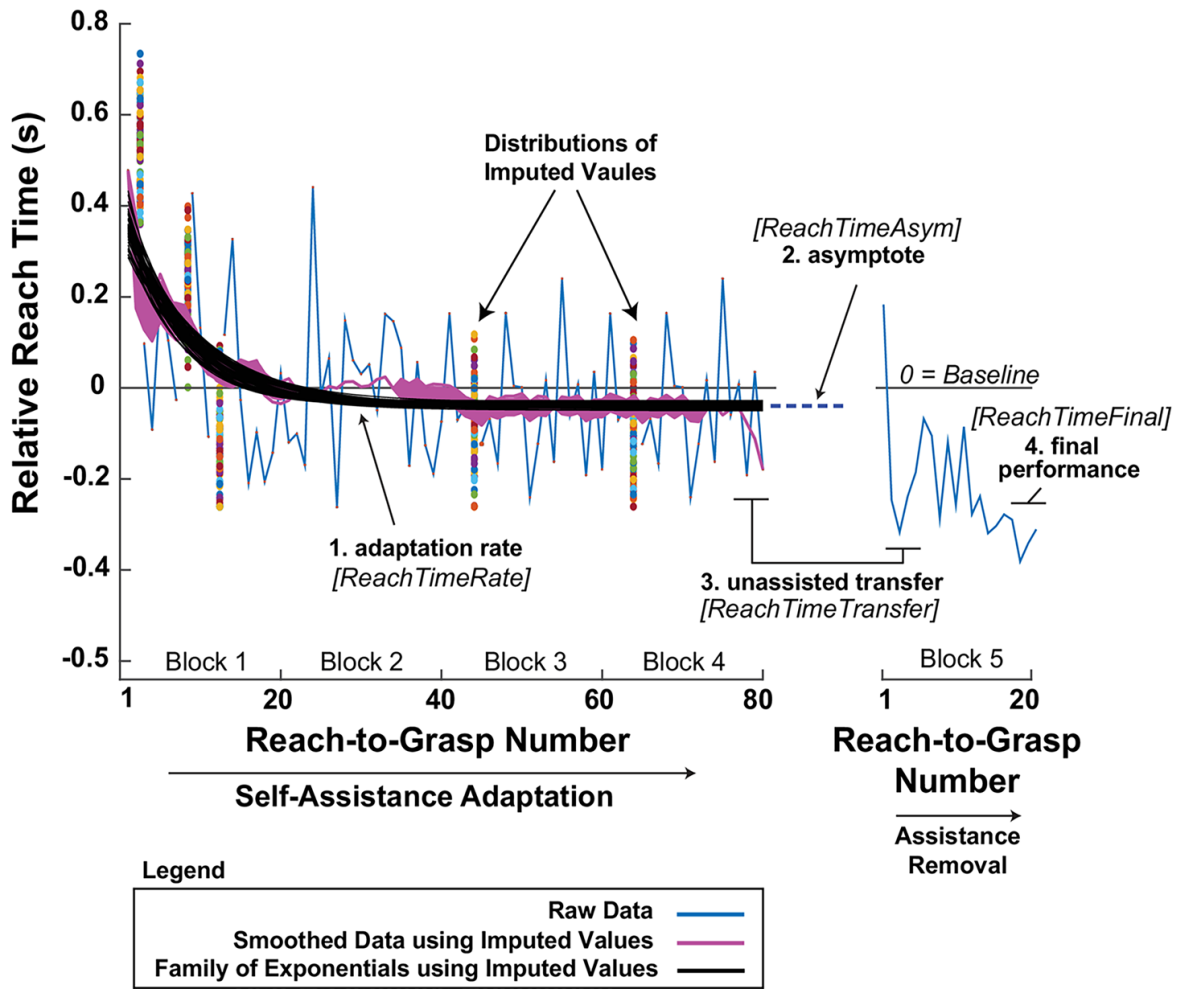
Example of exponential fitting for one participant in the self-assist group and other measures characterizing adaptation and learning. Missing data (failed reach-to-grasp attempts) were replaced using multiple imputation. In this example, each of the 5 failed reach-to-grasp attempts were imputed 100 times, creating 100 data sets. Each of the 100 data sets was smoothed (magenta curves), and an exponential was fit to each smoothed data set (black curves). This imputation process also shows the sensitivity of the fitted curves to the missing (imputed) values. This participant performed with relatively high variability between reach-to-grasp attempts

In this experiment, we focus on *ReachTime* because it specifically reflected performance during the phase of the task most affected by self-assistance, when participants could use the hEXO to resist the involuntary closure of the left hand from DFES as they attempt to reach for and grasp the object. *ReachTimeRate* was the primary dependent variable used to assess the rate of motor adaptation, defined as the exponential rate of change in the relative *ReachTime* across Blocks 1–4 for each participant (Fig. 6). However, before fitting exponential models, it was necessary to address missing *ReachTime* values resulting from failed reach-to-grasp attempts. The assist group experienced a higher proportion of failed reach-to-grasp attempts compared to the control group, averaging about 4% versus almost zero, respectively. Since the likelihood of missing data was related to group membership and could therefore bias the analysis if pointwise deletion was used, we used multiple imputation to replace the missing data. Multiple imputation preserves the inherent variability and uncertainty of missing data, which can help reduce bias and enhance the reliability of parameter estimates [53].

For the multiple imputation procedure, missing relative *ReachTime* values were replaced using linear interpolation from the nearest existing data points (via MATLAB’s *fillmissing* command), using the series of 80 reach-to-grasp attempts for each condition and participant. To account for uncertainty while preserving the natural variability of the data, random noise with a standard deviation equal to 50% of the global standard deviation of the observed data was added to the imputed values. This procedure was repeated independently for each of 100 imputations, resulting in 100 datasets for each participant, each incorporating different plausible estimates of missing values. Although 5–10 imputations are typically sufficient in practice, recent recommendations suggest using 20–100 imputations for improved precision and stability, particularly when computational time is not a limiting factor [54].

Each participant’s 100 imputed relative *ReachTime* datasets were smoothed using a 20 reach-to-grasp attempt moving average before fitting the exponential model. This filtering reduced noise and within-participant variability between reach-to-grasp actions, ensuring the exponential fit more accurately captured underlying adaptation trends. A sensitivity analysis showed that *ReachTimeRate* was sensitive to very short window sizes, and relatively insensitive for window sizes of 20 or greater; hence, a window size of 20 was chosen to prevent over-smoothing (see Supplementary Material for details). After smoothing, we used MATLAB’s “fit” function to fit exponential functions to the smoothed data, employing the Nonlinear Least Squares method (specifically, the Levenberg-Marquardt algorithm) with the robust fitting option (the least absolute residual method). Thus, each participant had 100 exponential fits. The fitted exponentials were of the form $\:ReachTime\:=\text{a}\cdot\:{\text{e}}^{\text{b}\cdot\:\text{x}}+\text{c}$, where $\:a$ is a coefficient that scales the exponential function, $\:\text{e}\:$is the base of the natural logarithm, 𝑏 is a coefficient that affects the rate of growth or decay, defined as *ReachTimeRate*, 𝑐 is the horizontal asymptote, and $\:\text{x}$ is the reach-to-grasp attempt number (Fig. 6), with initial conditions set to [$\:a$ = 0.5, $\:b$ = -0.1, and $\:c$ = -0.01], based on preliminary analysis. The goodness-of-fit was assessed by computing the coefficient of determination (R²) for the fit of each of the 100 imputed and smoothed data sets, averaging these 100 R² values for each participant, and then averaging across participants within each group to obtain a group-level estimate of the exponential’s goodness-of-fit to the relative *ReachTime* data.

One participant in the self-assist group failed to successfully complete the first two attempts. This participant also showed no discernible trend in relative *ReachTime* over practice. Multiple imputation showed that the adaptation rate for this participant was highly sensitive to the values of the first two missing points. For instance, the average standard deviation of *ReachTimeRate* across imputations for the other participants was 0.064, while the standard deviation for the outlier participant was 0.637 – an order of magnitude larger. Because of the lack of confidence in the exponential fitting for this participant, their adaptation rate was left undefined and not included in the analysis.

#### Secondary motor adaptation variables

Secondary variables of interest were the horizontal asymptote of the fitted exponentials, *ReachTimeAsym* (parameter *c*; #2 in Fig. 6), the degree of unassisted transfer, relative *ReachTimeTransfer* (#3 in Fig. 6), and the final no-assist performance level, relative *ReachTimeFinal* (#4 in Fig. 6). Unassisted transfer was assessed by computing the difference between the mean relative *ReachTime* of the last 5 self-assist reach-to-grasp actions during adaptation (Block 4) and the mean of the first 5 reach-to-grasp actions in the assistance removal block (Bock 5). The same difference was calculated for the control group, which was used for comparison, but in this case, both blocks were unassisted as these participants did not self-assist. Relative *ReachTimeFinal* was computed as the average relative *ReachTime* of the last 5 reach-to-grasp actions in the last no-assistance block while wearing the exoskeleton with DFES (Block 5).

#### Sensitivity analysis for exponential-derived motor adaptation variables

We tried other missing data handling methods to see how different approaches affect exponential-derived measures *ReachTimeRate* and *ReachTimeAsym*. This included testing the effects of single imputation, replacing failed trials with the maximum time-out time (2.0 s), pointwise deletion, and using regular vs. robust exponential fitting approaches (see Supplementary Material for details).

#### Other performance variables

As for Experiment 1, *PcntTaskSuc* and *PcntReachSuc* were calculated for each block of 20 reach-to-grasp-and-lift attempts. For relative *InitAper* and relative *PeakAper*, data inspection revealed that a few reach-to-grasp attempts were classified as extreme outliers (> 3 standard deviations from the mean), resulting from severe marker occlusions, and these were removed from the analysis (1 attempt for *InitAper* and 6 attempts for *PeakAper;* these are out of 1600 total attempts across all adaptation blocks for all participants). To further evaluate potential changes in movement execution, we also quantified the peak speed during reach-to-grasp (see Supplementary Material for methods and analysis details).

#### Quantifying self-assistance

For Experiment 2, one group of participants was allowed to self-assist using the exoskeleton. The amount of self-assistance was estimated from the recorded muscle activity of the unimpaired, assisting arm. Using 4th order dual-pass Butterworth filters, the raw EMG data were high-pass filtered (0.5 Hz cutoff) to remove any movement artifacts, rectified, and then low-pass filtered (4 Hz cutoff) to create linear envelopes. The resting amplitude of the linear envelopes, obtained during initial calibration, was subtracted to remove the signal amplitude due to sensor noise. Then, the linear envelopes were normalized to each participant’s maximum voluntary contraction (MVC). Finally, the linear envelopes were integrated over the reaching movement (from the reach start to achieving a 2 cm object lift), giving the integrated EMG (IEMG) for both the wrist/hand extensors, *ExtIEMG*, and the wrist/hand flexors, *FlexIEMG*. To provide more readily interpretable units, the IEMG was normalized by the reach duration (thus, the reported IEMG is mathematically equivalent to the average EMG). The IEMG variables included all attempts where a successful grasp was achieved, regardless of the final task outcome.

Technical issues related to the IEMG data collection resulted in missing data. The MVC data for one participant in the self-assist group was not saved correctly, and an equipment malfunction prevented EMG data collection from the wrist/hand flexors for one participant in the control group. Thus, these data were not included in the EMG analysis. A programming bug caused the last reach’s EMG data to be missed for each block for most participants (*n* = 15), and the first six participants to have an extra 21st reach on some attempts. To ensure that all participants had the same number of attempts analyzed, only the first 19 reaches of each block were included in the IEMG analysis. The first six participants had their EMG data sampled at a rate of 120 Hz instead of 1000 Hz by mistake (three in the self-assist group; three in the control group). Although under-sampling may have significant effects on measures related to muscle activity timing (e.g., onset latency or burst duration), the effects on average amplitude-related EMG measures are less severe [55]. To empirically test the effects of the under sampled IEMG, we repeated the IEMG analysis 10 times, and each time we randomly selected three participants from the six non-affected participants in the assist group and down sampled the raw EMG data from 1000 Hz to 100 Hz (approximating the 120 Hz sampling rate but allowing decimation of the data without needing additional interpolation). This analysis did not change the statistical inference outcomes; therefore, the under-sampled participants’ data were included.

### Statistics

#### Baseline adjustments

We checked if by chance the randomization of participants into Experiment 2 resulted in groups with different baseline performance during the Experiment 1 DFES + hEXO condition. For this analysis, each Experiment 1 variable (*ReachTime*, *TaskTime*, *PcntTaskSuc*, *PcntReachSuc*, *InitAper*, and *PeakAper*), was subjected to a two-tailed independent samples *t*-test to check for group differences in the baseline DFES + hEXO condition.

#### Motor adaptation and performance

The *ReachTimeRate* and *ReachTimeAsym* metrics were determined using multiple imputations to account for missing data, as described earlier, and the imputed datasets were assessed for normality. *ReachTimeRate* was normally distributed for the self-assist group, with moderate skewness (Lilliefors test, *p* = 0.362 and skewness = -1.02, averaged across imputations). However, *ReachTimeRate* exhibited a non-normal distribution in the control group with high negative skewness (Lilliefors test, *p* = 0.012 and skewness = -1.40, averaged across imputations). To normalize the data, *ReachTimeRate* was first inverted and shifted by subtracting each value from the largest value in the dataset and adding 0.01 to eliminate zero and negative values; the resulting values were then log-transformed. After this transformation, which was similarly performed for both groups, the control group’s *ReachTimeRate* was approximately normally distributed with reduced skewness (Lilliefors test, *p* = 0.432 and skewness = 0.561, averaged across imputations).

For the control group, *ReachTimeAsym* also had a non-normal distribution (Lilliefors test, *p* = 0.001 and skewness = -1.93, averaged across imputations). However, a standard log transformation was ineffective at reducing this skewness; however, after inverting and shifting by subtracting each value from the largest value in the dataset and adding 0.1 to eliminate zero and negative values, a Box-Cox transformation successfully normalized the control group *ReachTimeAsym* (Box-Cox λ = −0.572; Lilliefors test, *p* = 0.048, and skewness = 0.590, averaged across imputations). The same transformation was applied to *ReachTimeAsym* for the self-assist group.

The imputed datasets were screened for outlier participants using the same criteria as in Experiment 1 (values exceeding 1.5 times the IQR beyond the third quartile or below the first). On average, there were 0.94 and zero outliers for *ReachTimeRate* for the self-assist and control groups, respectively, and zero and 0.16 outliers for *ReachTimeAsym* for the self-assist and control groups, respectively. These values represent the mean number of outliers across the 100 imputed datasets; values less than 1.0 indicate that some imputations contained no outliers.

Rubin’s rules were used to combine the results (parameter estimates and their associated variances) across imputations, accounting for both within- and between-imputation variability when estimating statistical significance [56]. These rules provide a principled method for pooling mean group differences between the self-assist and control groups’ *ReachTimeRate* (or *ReachTimeAsym*) and their corresponding standard errors across multiple imputed datasets. This yields a single, combined mean difference with appropriately adjusted standard errors and degrees of freedom, ensuring that statistical inferences reflect the uncertainty introduced by imputing missing data. Group differences were evaluated using the pooled statistics. Statistical significance was assessed using a pooled *t*-test, giving a pooled test-statistic, $\:{t}_{pooled}$, with degrees of freedom adjusted via the Barnard–Rubin small-sample correction. Hedges’ *g* was used to estimate effect sizes, correcting for small-sample bias. Full details of the pooling procedures are provided in the Supplementary Material.

*ReachTimeTransfer* and relative *ReachTimeFinal* did not rely on exponential fitting and did not require imputation. These variables had normal distributions and there were no outliers. One sample *t*-tests were performed separately for the self-assist and control groups for *ReachTimeTransfer*, based on the difference between the average relative reach time for the last five reach-to-grasp attempts in Block 4 and the first five reaches in Block 5. A two-tailed independent samples *t*-test comparing the groups was performed for *ReachTimeFinal* using Satterthwaite’s approximation for the effective degrees of freedom due to unequal variances between the groups (Levene’s test *p* < 0.05).

#### Reach and task success

The percentage of task attempts with successful reach-to-grasp-and-lifts (*PcntTaskSuc)* and the percentage of successful reach-to-grasps (*PcntReachSuc*) were calculated for each adaptation block of 20 attempts. These variables had non-normal distributions and a high negative skew (average *PcntTaskSuc* skewness across blocks= -1.38 and − 2.66 for the self-assist and control groups, respectively; average *PcntReachSuc* skewness across blocks = -0.74 and − 2.67 for the self-assist and control groups, respectively; skewness measures were averaged across the adaptation blocks). For *PcntTaskSuc*, following a log transformation for outlier checks (using the higher 3 × IQR threshold for bounded percentage data), two data points were excluded from the self-assist group (out of 40 data points total across the four blocks) and two data points were excluded from the control group (out of 40). For *PcntReachSuc*, there were no outliers for the self-assist group, but eight out of 40 data points were removed from the control group. Most participants in the control group had 100% success. The untransformed data with outliers removed was then used for analysis.

Because the success rate variables (*PcntTaskSuc* & *PcntReachSuc*) were negatively skewed, we used GLMMs with a gamma distribution and log-link. The gamma distribution requires positively skewed data with positive, nonzero values. Thus, we transformed the data by subtracting the percentages from 100 and adding a nominal value of 0.1% to any zero data points. The GLMMs included group as a between-subjects factor (self-assist or control), time as a within-subjects factor with repeated measures (four time points), and participant ID as a random factor. A diagonal covariance structure was used because the autoregressive covariance structure did not significantly improve the AIC and BIC for *PcntTaskSuc*, and the model failed to converge for *PcntReachSuc*, but did converge with the diagonal structure.

For each group, unassisted transfer was defined as the difference in *PcntTaskSuc* and *PcntReachSuc* between the Block 4 (with assistance for self-assist group only) and Block 5 (no self-assistance for both groups). One-sample Wilcoxon signed-rank tests were used to test whether the medians differed from a hypothesized value of zero (i.e., no difference between Blocks 4 and 5). Finally, unassisted transfer was assessed for between-group differences using two-sample Wilcoxon ranked-sum tests.

#### Hand aperture and self-assistance

An analysis of the left-hand aperture and right forearm IEMG (a proxy for self-assistance) measures did not show exponential trends across the four self-assistance reach-to-grasp and object lift blocks (Blocks 1–4), as was observed for relative *ReachTime*. Thus, we averaged these measures across each practice block for each participant, giving one data point for each subject per block. The participant data within each block was tested for normality, separately for the two groups, using the Lilliefors test. For *InitAper* and *PeakAper*, the participant data within nearly all blocks were normally distributed, with the exception of one block showing mild deviation from normality (*p* = 0.038). However, *ExtIEMG* and *FlexIEMG* had non-normal distributions (*p* < 0.05) with a positive skew, so these measures were log-transformed. Since the IEMG measurements included zero values, which occurred when the IEMG was below the noise threshold, a small constant (0.01%) was added to any zero values prior to the log transformation.

For each group, the aperture data were screened for outliers across blocks using the IQR method: values exceeding 1.5 times the IQR beyond the third quartile or below the first quartile. For *InitAper*, three outlier participant blocks were removed from the control group (out of 40 blocks total: 4 blocks x 10 participants) and four were removed from the assist group (also out of 40 blocks total). For *PeakAper*, six outlier participant blocks were removed for the control group and four were removed for the assist group (out of 40 blocks total). No outliers were detected for the log-transformed *ExtIEMG* and *FlexIEMG* for the self-assist and control groups.

For statistical analysis of IEMG and aperture measures, we used GLMMs with a gamma distribution and a log link with a first-order autoregressive covariance structure. These models included group as a between-subjects factor (self-assist or control), time as a within-subjects factor with repeated measures (Blocks 1–4), and participant ID as a random factor. For the aperture measures, GLMMs were used with a linear model structure and diagonal covariance structure (the autoregressive covariance structure did not improve AIC or BIC).

To assess unassisted transfer for *InitAper*,* PeakAper*,* ExtIEMG*, and *FlexIEMG*, one-sample *t*-tests were performed for the self-assist group, using the difference between the average relative reach time for the last five reaches in the adaptation series (Block 4) and the first five reaches in the subsequent unassisted transfer block (Block 5). Similar tests were performed for the control group, although here transfer is not assessed because the control group performs the same condition in Blocks 4 and 5.

The aforementioned GLMM analysis would obscure any rapid changes occurring in the first few reach-to-grasp attempts (i.e., nonlinearities in the data) in any given practice block. Thus, a within-block analysis was performed for Blocks 1–4. For each group and block, for *InitAper*, *PeakAper*, *ExtIEMG*, and *FlexIEMG*, the difference was taken for each participant between the average of the first 5 reaches and the average of the last 5 reaches. Outlier participants within groups were identified using our standard criteria (> 1.5 IQR) for each practice block. For *ExtIEMG*, two outlier participant blocks were removed from the control group and two were removed from the assist group (out of 40 blocks total per group). For *FlexIEMG*, four outlier blocks were removed from the control group and two from the assist group. For *InitAper*, seven outlier participant blocks were removed from the control group, and five were removed for the assist group (out of 40 blocks total per group). For *PeakAper*, four outlier blocks were removed from the control group and four from the assist group.

For each within-block difference measure, one-sample *t*-tests were performed. To adjust for multiple comparisons and control the family-wise error rate, we employed the Westfall-Young permutation method (with 10,000 permutations) to generate an empirical distribution of test statistics under the null hypothesis. For each permutation, we recalculated the test statistics. The adjusted *p*-values were then derived by comparing the observed test statistics to the empirical distribution, thus helping control for Type I errors while accounting for dependencies among tests.

## Results (Experiment 1)

### Motor performance

**Fig. 7:**
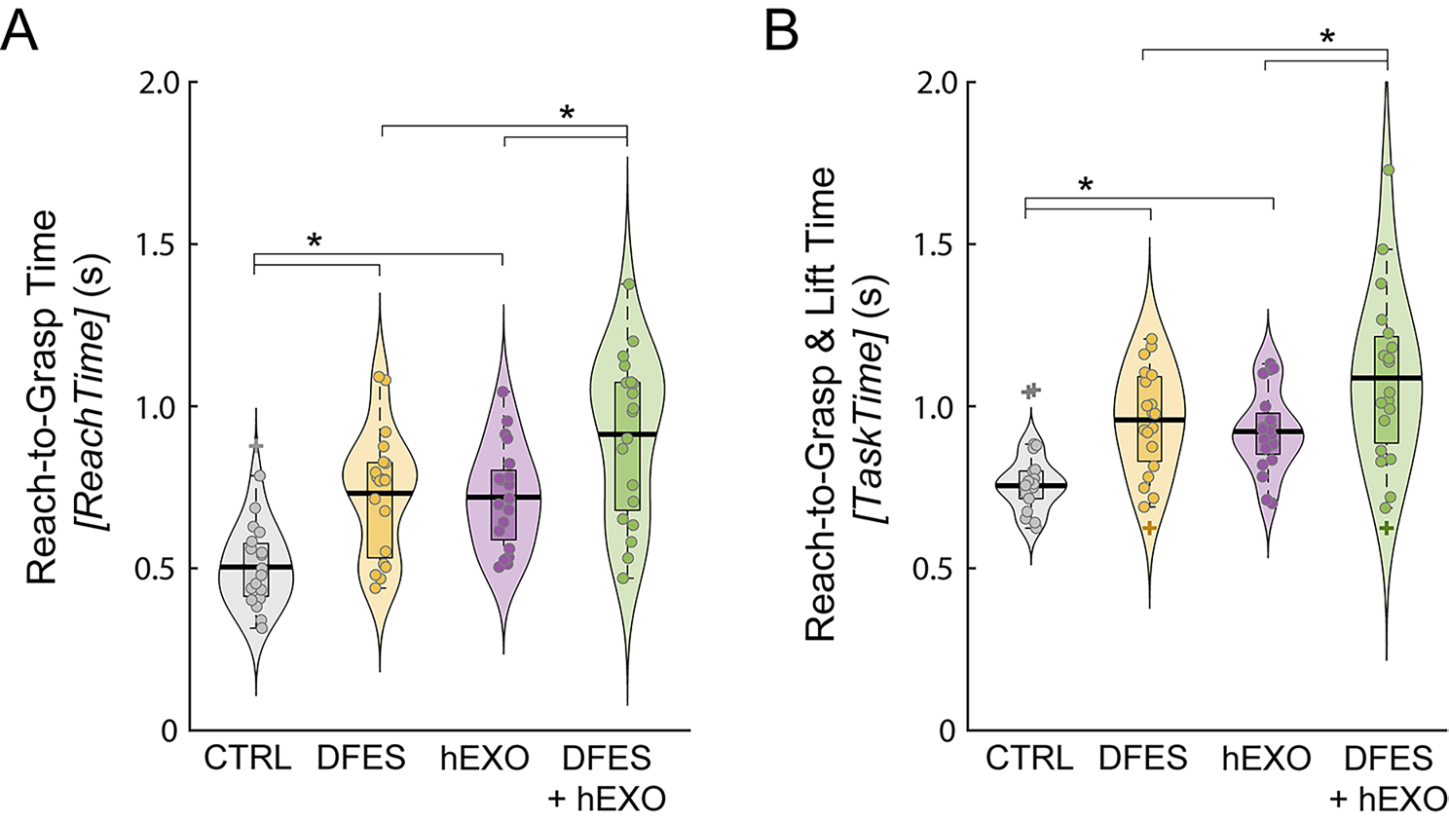
Average reach-to-grasp time (**A**) and reach-to-grasp & lift time (**B**) for each condition in Experiment 1 (DFES: receiving dysfunctional electrical stimulation; hEXO: wearing hydrostatic exoskeleton; CTRL: without DFES and hEXO). Violin plots show probability density and boxplots show median (heavy horizontal bar) and interquartile range, excluding outliers. Dots are individual participants. +Outliers (more than 1.5 times the interquartile range above the third quartile or below the first quartile). *Bars above plots show significant pairwise differences (*p* < 0.05)

Reach-to-grasp took longer when receiving DFES and/or wearing the hEXO (Fig. 7A). For *ReachTime*, there were main effects of DFES, *F*(1,75) = 45.0, *p* < 0.001, and hEXO, *F*(1,75) = 39.9, *p* < 0.001. There was no interaction between DFES and hEXO, *F*(1,75) = 0.176, *p* = 0.676. Pairwise comparisons showed that the DFES-only and hEXO-only conditions had a longer *ReachTime* than CTRL, and the combined DFES + hEXO condition had a longer *ReachTime* than the DFES-only and hEXO-only conditions (*p* < 0.001 for each comparison).

It took longer to complete the whole reach-to-grasp and object lift task when receiving DFES and/or wearing the hEXO (Fig. 7B). For *TaskTime*, there were main effects of DFES, *F*(1,72) = 27.8, *p* < 0.001, and hEXO, *F*(1,72) = 18.8, *p* < 0.001. Receiving DFES or wearing the hEXO increased the time it took to complete the entire task. There was no interaction between DFES and hEXO, *F*(1,72) = 0.136, *p* = 0.713 (Fig. 7). Pairwise comparisons showed that the DFES-only and hEXO-only conditions had a longer *TaskTime* than CTRL (both *p* < 0.001), and the combined DFES + hEXO condition had a longer *TaskTime* than the hEXO-only condition (*p* = 0.006) and the DFES-only condition (*p* = 0.038).

### Success rates

**Fig. 8:**
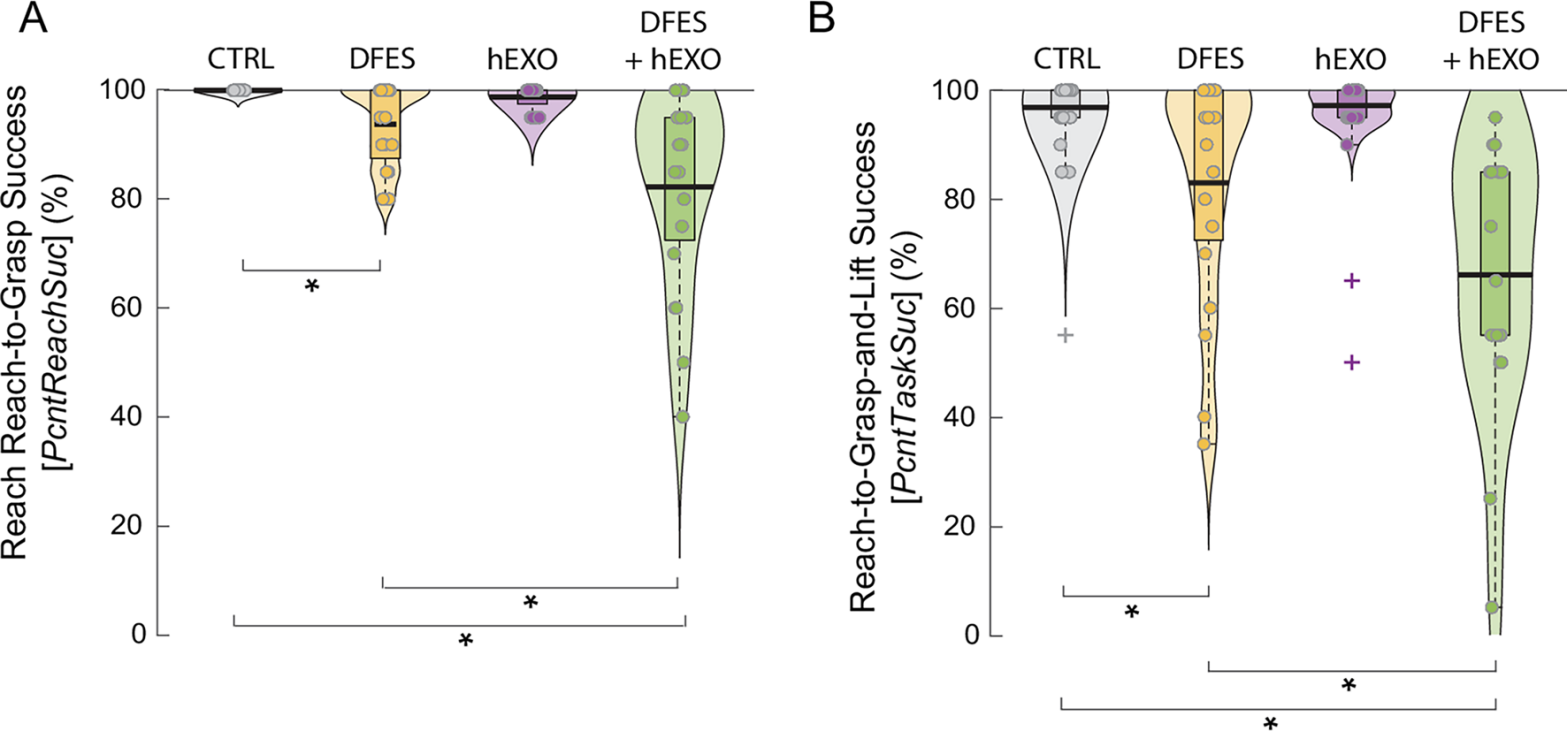
The percentage of successful reach-to-grasp actions (**A**) and the percentage of successful reach-to-grasp-and-lift actions (**B**) for each condition in Experiment 1 (DFES: receiving dysfunctional electrical stimulation; hEXO: wearing hydrostatic exoskeleton; CTRL: without DFES and hEXO). Violin plots show probability density and boxplots show median (heavy horizontal bar) and interquartile range, excluding outliers. Dots are individual participants. +Outliers (more than 3 times the interquartile range above the third quartile or below the first quartile). *Bars show significant pairwise differences (*p* < 0.05)

DFES reduced the likelihood of achieving a functional grasp, and this reduction was exaggerated when wearing the hEXO (Fig. 8A). For *PcntReachSuc*, there was an interaction between DFES and hEXO, *F*(1,74) = 7.47, *p* = 0.008. Receiving DFES reduced the likelihood of successfully reaching to grasp the object, and the success rate dropped substantially when the hEXO was worn in addition to receiving DFES. There were also main effects of DFES, *F*(1,74) = 168, *p* < 0.001, and hEXO, *F*(1,74) = 46.4, *p* < 0.001, with both effects lowering *PcntReachSuc*.

DFES reduced the likelihood of completing the task successfully and this reduction was exaggerated when wearing the hEXO (Fig. 8B). For *PcntTaskSuc*, there was an interaction between DFES and hEXO, *F*(1,74) = 4.49, *p* = 0.037. Receiving DFES reduced the likelihood of successfully completing the entire task, and the task success rate decreased further when the hEXO was worn in addition to receiving DFES. There were also main effects of DFES, *F*(1,74) = 29.7, *p* < 0.001, and hEXO, *F*(1,74) = 6.45, *p* = 0.013, with both effects lowering *PcntTaskSuc*.

### Hand aperture

**Fig. 9:**
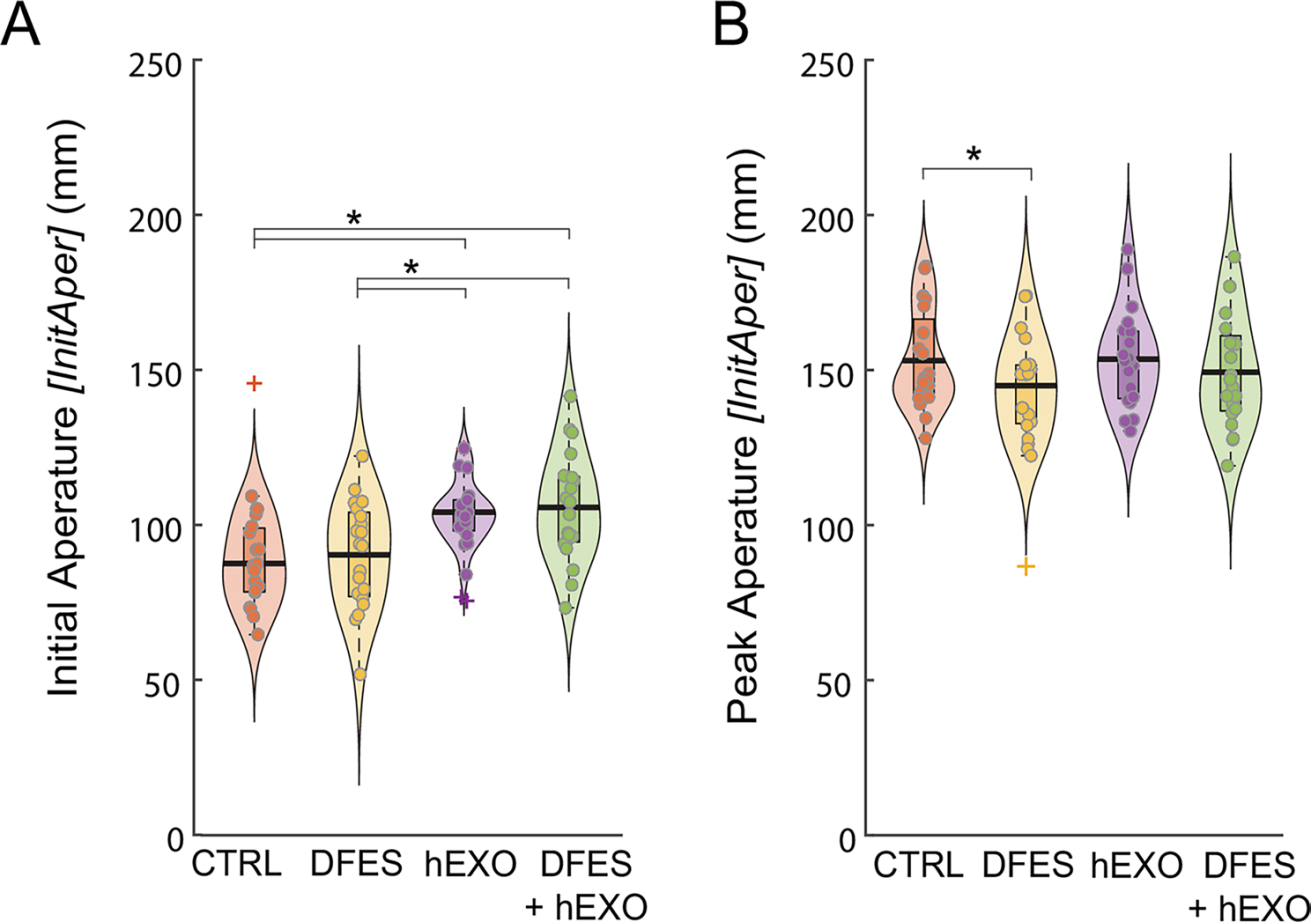
Average initial hand aperture (**A**) and peak hand aperture (**B**) for each condition in Experiment 1 (DFES: receiving dysfunctional electrical stimulation; hEXO: wearing hydrostatic exoskeleton; CTRL: without DFES and hEXO). Violin plots show probability density and boxplots show median (heavy horizontal bar) and interquartile range, excluding outliers. Dots are individual participants. +Outliers (more than 1.5 times the interquartile range above the third quartile or below the first quartile). *Bars show significant pairwise differences (*p* < 0.05)

Wearing the hEXO increased the initial hand aperture (Fig. 9A). For *InitAper*, there was a main effect of hEXO, *F*(1,73) = 31.0, *p* < 0.001, such that the initial left-hand aperture was larger in conditions involving the hEXO. There was no effect of DFES, *F*(1,73) = 0.893, *p* = 0.348 and no interaction, *F*(1,73) = 0.013, *p* = 0.909.

DFES reduced the peak hand aperture (Fig. 9B). For *PeakAper*, there was a main effect of DFES, *F*(1,76) = 5.37, *p* = 0.023, such that the peak left-hand aperture was smaller in conditions involving DFES. There was no effect of hEXO, *F*(1,76) = 0.823, *p* = 0.367 and no interaction, *F*(1,76) = 0.523, *p* = 0.472.

## Results (Experiment 2)

### Baseline checks

The control and self-assist groups had similar baseline performance. The Experiment 2 performance measures were normalized to the average performance in the DFES + hEXO condition in Experiment 1. For this condition, there were no differences between the groups for *ReachTime*, *t*(18.0) = 0.685, *p* = 0.502, *PcntTaskSuc*,* t*(18.0) = -0.151, *p* = 0.881, *PcntReachSuc*,* t*(18.0) = 0.557, *p* = 0.584, *InitAper*,* t*(18.0) = -0.345, *p* = 0.734, *or PeakAper*,* t*(18.0) = -0.508, *p* = 0.618, indicating that normalization based on this condition was unbiased.

### Adaptation to artificial impairment and self-assistance

**Fig. 10:**
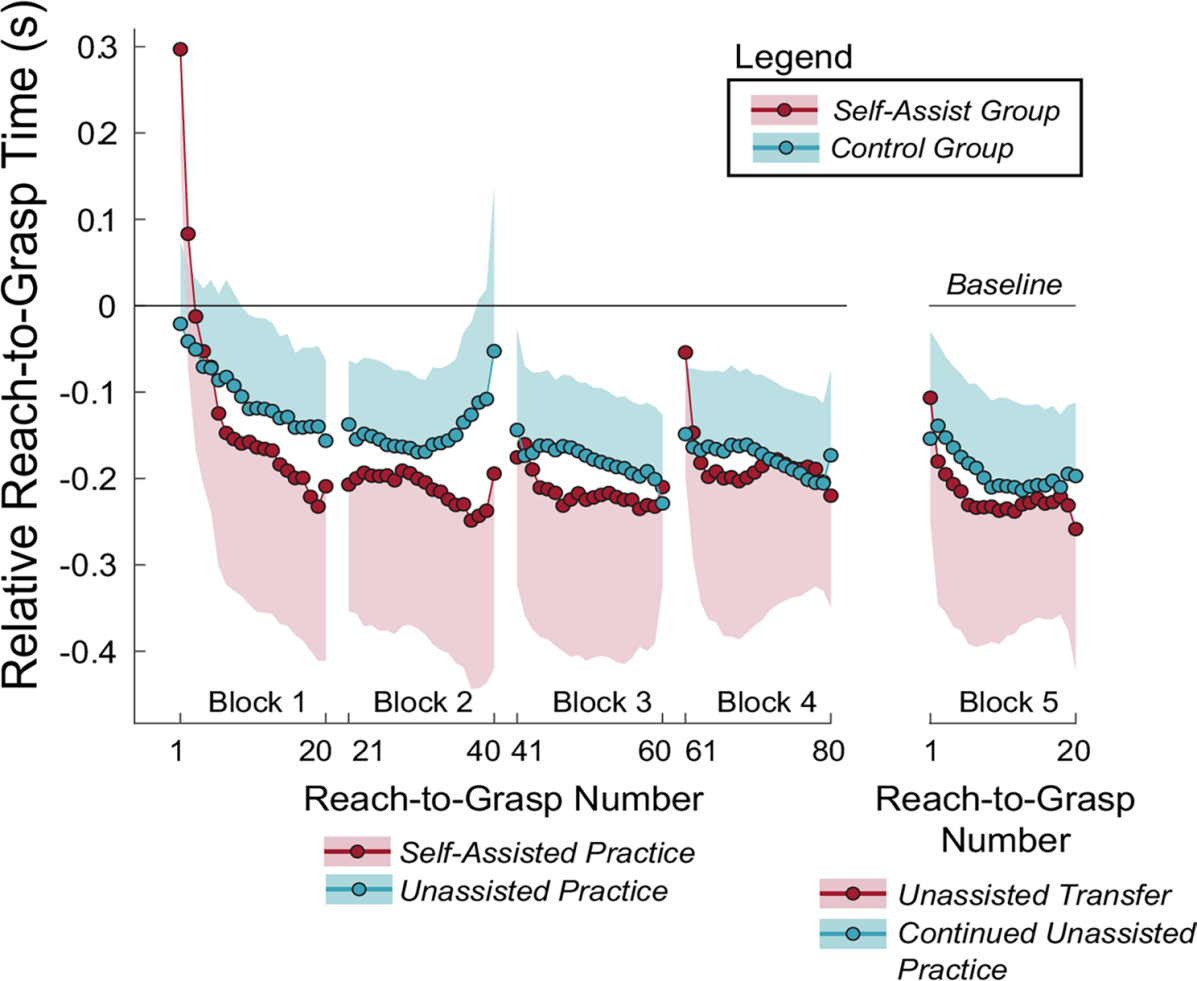
Relative reach-to-grasp times for the self-assist and control groups in Experiment 2. The lines with points across practice show the mean reach-to-grasp time across participants, and the shading shows one-half of the 95% confidence interval. The averaged data were smoothed with a 10-point moving average within blocks to better visualize trends. In Block 5, the ability to self-assist was removed for the self-assist group, while the control group continued practicing unassisted. The self-assist data does not include data from one outlier participant who had a very low rate of performance improvement across the practice session

**Fig. 11:**
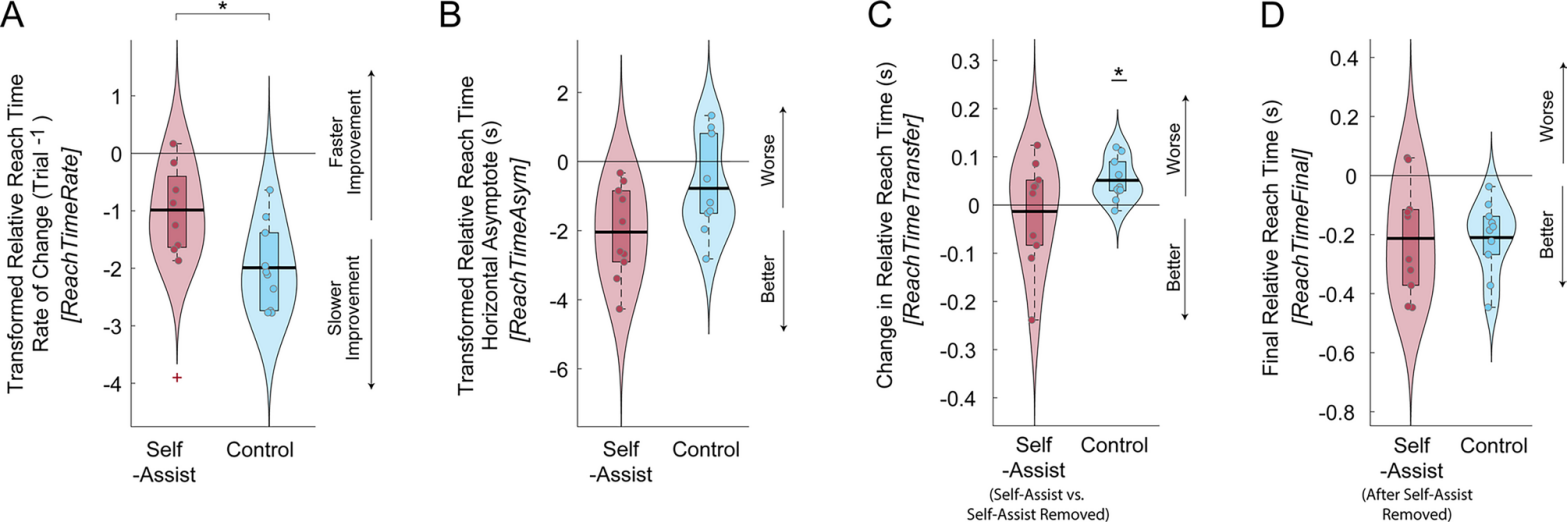
**A-B**: Log-transformed reaching time adaptation rate (**A**) and the Box-Cox transformed horizontal asymptote (**B**), based on exponential fits to each participant’s relative reach time (relative to an initial no-assist baseline) in the self-assist (red) and control (blue) groups for Experiment 2 (*significant difference between groups). **C-D**: Change in reach time between the last practice block with assistance (for the self-assist group) and the beginning of the next practice block where self-assistance was unavailable (**C;** *significantly different from zero) and final relative reach time achieved at the end of the last no-assistance practice block (**D**). Violin plots show probability density and boxplots show median (heavy horizontal bar) and interquartile range. Dots are individual participants. For the rate and asymptote variables (A & B), the value for each participant represents the mean values derived across 100 imputed data sets to replace missing data due to failed reaches during the exponential fitting. +Outliers (more than 1.5 times the IQR above the third quartile or below the first quartile)

Participants who could self-assist improved their reach-to-grasp time more quickly than the control group, who could not self-assist (Figs. 10 and 11A, amp and B). A statistical analysis of the log-transformed reach-to-grasp adaptation rate constants (*ReachTimeRate*) showed that the self-assist group improved significantly faster than the no-assist group, $\:{t}_{pooled}$(80.91) = 2.71, SE = 0.364, *p* = 0.008, and Hedges’ g = 1.26, indicating a large effect size. As shown in Fig. 10, for the self-assist group, there was an increase in relative *ReachTime* at the start of practice compared to baseline, but this increase was brief, lasting only two reach-to-grasp attempts. However, the self-assist group’s relative *ReachTime* quickly improved, outperforming the control group until it equalized later in practice. A supplemental analysis of the peak reach-to-grasp speed echoed these results, with a brief decrease in peak movement speed on the first few reach-to-grasp attempts for the self-assist group, followed by a rapid improvement (see Supplemental Material for details). The self-assist group also exhibited a lower relative reach time asymptote (*ReachTimeAsym*), suggesting better performance after the decay of adaptation transients, but the difference did not reach statistical significance, $\:{t}_{pooled}$ (62.9) = -1.91, SE = 0.637, *p* = 0.060, and Hedges’ g = -0.858.

The sensitivity analysis showed that the group differences in *ReachTimeRate* remained significant across missing data handling approaches, including single and multiple imputation, as well as time-out replacement (see the Supplementary Material for full results). In contrast, pointwise deletion yielded non-significant results (*p* = 0.115 and *p* = 0.146 for regular and robust fitting, respectively), likely due to reduced power from disproportionately excluding more trials from the self-assist group who more frequently timed-out on attempts, and poorer exponential model fits, where the average R^2^ was 0.52 compared to 0.84 for multiple imputation. For *ReachTimeAsym* the group differences were non-significant using the multiple imputation and pointwise deletion approach, but significant when using time-out and single imputation (conclusions are based on the results of the multiple imputation approach).

### Unassisted transfer and final performance

There was no performance decrement when self-assistance was removed, and both groups achieved the same level of unassisted reach-to-grasp performance (Fig. 11C&D). For the self-assist group, the relative reach time did not change when the ability to self-assist was removed, i.e., *ReachTimeTransfer* was not different from zero, *t*(9.0) = -0.382, *p* = 0.712 (Fig. 11C). However, *ReachTimeTransfer* was different from zero for the no-assist group, *t*(9.0) = 3.72, *p* = 0.005, reflecting a small decrement in performance (n.b., since this is the control group, the conditions were the same, i.e., there was no self-assistance to remove). There was no difference between the groups for the final performance level achieved without self-assistance, as quantified by relative *ReachTimeFinal*, *t*(18.0) = -0.039, *p* = 0.969 (Fig. 11D). We also examined the peak reach-to-grasp speed but found no significant group differences or changes following assistance removal (see Supplementary Material for details).

### Success rates

**Fig. 12:**
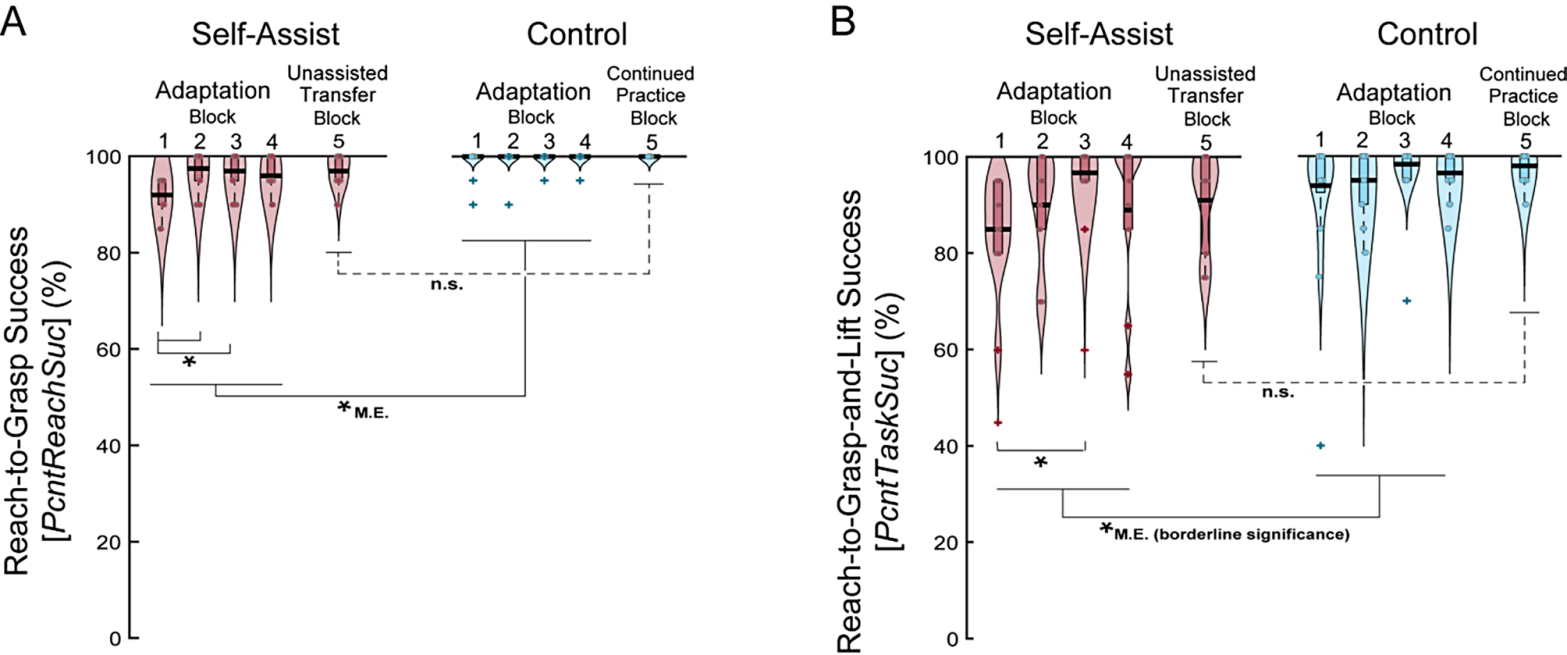
Percentage of successful reach-to-grasp (**A**) and reach-to-grasp-and-lift attempts (**B**) for the self-assist and control groups in Experiment 2. Violin plots show probability density and boxplots show median (heavy horizontal bar) and interquartile range (shaded box). Dots are individual participants. +Outliers (more than 3 times the interquartile range above the third quartile or below the first quartile). *Significant pairwise difference (*p* < 0.05). *M.E = significant main effect of group (*p* < = 0.05). n.s. = no significant difference (*p* > 0.05)

The self-assist group had more reach-to-grasp failures than the control group while adapting to self-assistance, and there was no change in reach success when assistance was removed. Both groups improved reach success with practice and finished with similar rates of reach success at the end of practice (Fig. 12A). For PcntReachSuc, there was a significant group x time interaction, *F*(3,64) = 3.12, *p* = 0.032, based on the GLMM results. The self-assist group showed improvements from Block 1 to Block 2 (*p* < 0.001), followed by a performance plateau (Blocks 2 vs. 3: *p* > 0.05). The control group maintained high success throughout practice. A main effect of group, *F*(1,64) = 99.0, *p* < 0.001, reflected an overall greater percentage of reach-to-grasp failures in the self-assist group. A main effect of time, *F*(3,64) = 3.12, *p* = 0.032, reflected the overall improvement across all participant’s success rates with time. The one-sample Wilcoxon signed-rank test for assessing unassisted transfer between Blocks 4 and 5 showed that, after assistance was removed, performance remained stable in the self-assist group (*W* = 10.00, *p* = 0.750), as well as for the control group between the final two unassisted practice blocks (*W* = 0.00, *p* = 1.00). The two-sample Wilcoxon rank-sum test showed no difference between groups in their grasping success during the final no-assistance block (Block 5) (*W* = 96.0, *p* = 0.059).

For full task success (requiring successful reach, lift, timing, and object angle), the self-assist group initially showed a slightly lower overall success rate. However, both groups improved with practice, and the self-assist group maintained their performance even after assistance was removed. By the end of practice, task success rates were comparable between groups (Fig. 12B). For *PcntTaskSuc*, a borderline-significant group effect was observed, *F*(1,68) = 4.00, *p* = 0.050, based on the GLMM results, with the self-assist group exhibiting more task failures. There was a significant time effect, signifying improvement over time, *F*(3,68) = 4.45, *p* = 0.007, but no group x time interaction, *F*(3,68) = 1.23, *p* = 0.304. Pairwise comparisons showed the self-assist group improved from Block 1 to Block 3 (*p* = 0.009). As with reach success, the one-sample Wilcoxon signed-rank test for assessing unassisted transfer between Blocks 4 and 5 showed that removing assistance did not impact task success for the self-assist group (*W* = 18.5 *p* = 0.516). The control group also had no difference between Block 4 and 5 (*W* = 7.00, *p* = 0.625). There was no difference between the groups in *PcntTaskSuc* for the final no-assistance practice block (Block 5) (*W* = 128, *p* = 0.071).

### Self-assistance

**Fig. 13:**
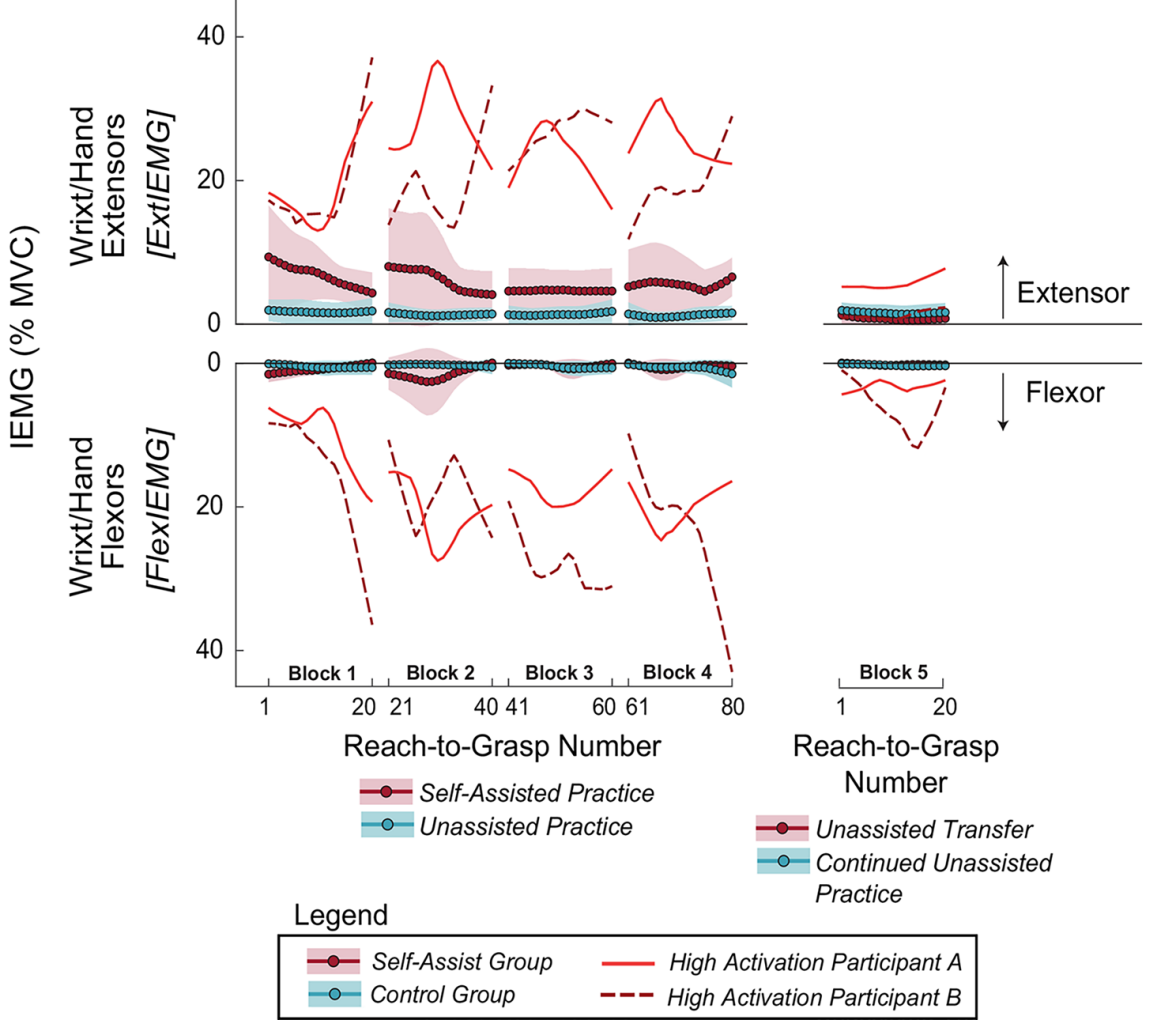
Integrated muscle activity in wrist extensors (top) and flexors (bottom) during the reach-to-grasp, normalized to a maximum voluntary contraction (IEMG). The lines and points show the mean across participants, and the shading shows the 95% confidence interval. Two participants in the self-assist group who demonstrated high antagonistic co-activation are separated out and shown as dashed and solid red lines. The averaged data were smoothed with a 10-point moving average within blocks to better visualize trends. In Block 5, the ability to self-assist was removed from the self-assist group, while the control group just continued to practice unassisted

The self-assist group assisted themselves, maintained a similar level of self-assistance throughout practice, and stopped self-assisting once the mechanical coupling was removed (Fig. 13). Self-assistance was estimated from IEMG of the assisting forearm, with greater extensor activation relative to flexor activation interpreted as increased assistive effort. The self-assist group had significantly higher *ExtIEMG* than the control group across the adaptation blocks (Blocks 1–4), *F*(1,68) = 15.1, *p* < 0.001. There was no effect of time, *F*(3,68) = 1.39, *p* = 0.139, and no group x time interaction, *F*(3,68) = 0.061, *p* = 0.980. There was no difference in *FlexIEMG* between groups, *F*(1,63) = 3.75, *p* = 0.057, nor a significant effect of time, *F*(3,63) = 2.37, *p* = 0.079, or interaction *F*(3,63) = 1.52, *p* = 0.219.

Two participants in the self-assist group showed elevated activity in both flexors and extensors, i.e., high antagonistic coactivation. Although they were not statistical outliers, they are visualized separately in Fig. 13. Removing these participants maintained the *ExtIEMG* group effect. However, when excluding these participants for *FlexIEMG*, the model did not converge using the autoregressive covariance structure, but did converge with a diagonal structure, with a newly significant main effect of time, *F*(3,55) = 8.72, *p* < 0.001. In this case, pairwise contrasts showed that *FlexIEMG* decreased after Block 1 (*p* < 0.01), suggesting early adaptation. Within-block comparisons (first vs. last five attempts) showed no significant changes in IEMG for either muscle group, group, or block (all *p* > 0.05).

To assess the effect of removing self-assistance, we compared IEMG between Blocks 4 and 5. In the self-assist group, *ExtIEMG* significantly decreased when the mechanical coupling between the hands (via the hEXO) was removed, *t*(8) = 2.63, *p* = 0.030. No such change was observed in the control group, *t*(9) = -1.25, *p* = 0.243, who were never mechanically coupled with the hEXO. *FlexIEMG* did not differ between Blocks 4 and 5 for either group (self-assist: *t*(8) = 1.44, *p* = 0.189; control: *t*(8) = 1.45, *p* = 0.185). Excluding high-coactivation participants did not change any of these outcomes.

### Hand aperture

**Fig. 14:**
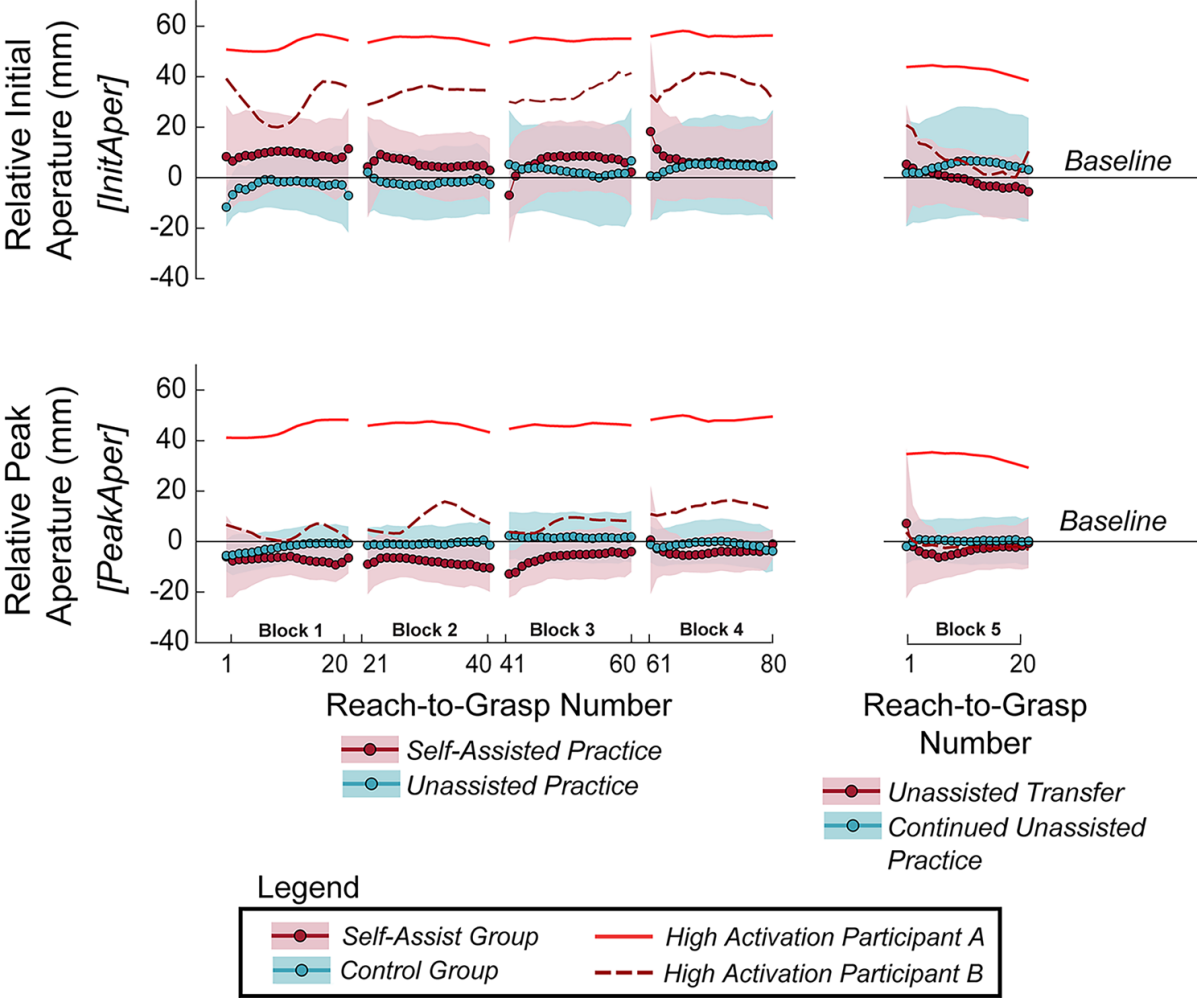
Initial (top) and peak (bottom) hand aperture during the reaching action, relative to the baseline condition. The lines and points show the mean across participants, and the shading shows the 95% confidence interval. Two participants in the self-assist group who demonstrated high antagonistic co-activation are separated out and shown as dashed and solid red lines. The averaged data were smoothed with a 10-point moving average within blocks to better visualize trends. In Block 5, the ability to self-assist was removed from the self-assist group, while the control group just continued to practice unassisted

Hand aperture did not differ between groups, remained stable over time, and was unaffected by the removal of assistance (Fig. 14). Initial and peak aperture values during reach-to-grasp were comparable between the self-assist and control groups across all four adaptation blocks, with no significant changes over time. For *InitAper*, normalized to the DFES + hEXO condition for Experiment 1, there were no effects of group, *F*(1,65) = 3.29, *p* = 0.074, time, *F*(3,65) = 0.422, *p* = 0.738, or group x time, *F*(3,65) = 0.472, *p* = 0.703 (Fig. 14; top). For *PeakAper*, results were similarly non-significant for group, *F*(1,76) = 0.946, *p* = 0.334, time, *F*(3,62) = 0.615, *p* = 0.608, and group x time, *F*(3,62) = 1.28, *p* = 0.291 (Fig. 14; bottom).

Within-block comparisons (first vs. last five reach-to-grasp attempts within a block) showed no significant changes in *InitAper* and *PeakAper* for either group across any of the four adaptation blocks (all *p* > 0.05), indicating stable hand shaping during individual practice sessions. To evaluate unassisted transfer, we compared Blocks 4 and 5. No changes were found in *InitAper* (self-assist: *t*(9) = 1.19, *p* = 0.265; control: *t*(9) = 1.02, *p* = 0.334) or *PeakAper* (self-assist: *t*(9) = 0.523, *p* = 0.613; control: *t*(9) = -1.92, *p* = 0.087). Repeating the analysis without the two high-coactivation participants yielded the same statistical conclusions, despite these individuals also having a larger hand aperture, highlighted in Fig. 14 for reference.

## Discussion

### Motor adaptation to self-assistance

Participants with a temporary neuromotor impairment who used a passive, bilateral, hydrostatic exoskeleton (hEXO) improved their reach-to-grasp performance more rapidly than controls who practiced without self-assistance, in line with the main hypothesis of this study. Manual self-assistance was associated with a brief initial performance decrement, lasting only two reach-to-grasp attempts on average; however, this decrement was offset by the comparably more rapid improvement of the self-assist group. Both groups ultimately reached the same level of performance by the end of practice, and the self-assist group maintained their gains after their ability to self-assist was removed. This indicates that participants did not become reliant on their assisting arm to retain their improvements. Although self-assistance led to a slight increase in the likelihood of failing to complete the reach-to-grasp portion of the task within the prescribed 2.0 s time limit—about one failure in every 20 attempts—both groups achieved similar success rates in the final unassisted practice block. These findings suggest that manual self-assistance can hasten motor improvement without inducing maladaptive dependence, supporting its potential value in rehabilitation settings.

To test our self-assistance hypothesis, we employed a relatively complex and intentionally demanding real-world, functional motor task. Participants used their non-dominant hand to perform a reach-to-grasp and object lift task while experiencing an artificial impairment, which disrupted hand opening. They were instructed to perform the task as quickly as possible while meeting spatial accuracy constraints on the object’s final position. This experimental design enabled us to examine whether self-assistance could promote motor improvement under challenging sensorimotor conditions and allowed us to test an alternative hypothesis: that the added bimanual coordination demands of self-assistance might negate any performance benefits during complex tasks. The artificial impairment effectively increased task difficulty by reducing accuracy, with only 65% grasp success, and slowing performance time, with a 50% increase in reach-to-grasp time. This impairment created a meaningful space for improvement, allowing us to test our self-assistance hypothesis in a model system of neuromuscular impairment.

### Bimanual coordination demands during self-assistance

The self-assist group had a small decrease in the reach-to-grasp and overall task success rates; however, this would in part be predicted by their slightly faster completion times and the speed-accuracy tradeoff [57]. The requirement to learn how to coordinate self-assistive actions could have also induced a more exploratory strategy with a willingness to tolerate more errors. Nevertheless, the self-assist group did not appear severely hindered by the added challenge of coordinating both hands. This is notable given that self-assistance introduces bimanual control demands that could reasonably impair performance, especially in early practice. Whole-arm sequence learning may be impacted by differences in reach control between the dominant and non-dominant arms [58], suggesting that bimanual tasks may impose asymmetric control challenges. These findings suggest that healthy individuals can adapt to the bimanual control demands of self-assistance without substantial performance impairments, even when coordinating two limbs with different control demands (e.g., giving vs. receiving assistance).

It is possible that self-assistance promoted participants’ sense of autonomy and improved their confidence, engagement, or persistence during the early learning phase. Attention may further shape this coupling: de Poel et al. [59] found that focusing attention on one limb, such as the non-dominant hand, can bias bimanual coordination dynamics in its favor. Coordinating the arms in a symmetric, in-phase grasping task may engage neural and perceptual mechanisms that facilitate unified control and adaptive processes. For example, bimanual reaching and grasping reduces intracortical inhibition and enhances control of an impaired arm by making indirect pathways from the ipsilateral hemisphere more accessible [26, 60]. These effects are not seen with unilateral training, which has instead been linked to increased ipsilateral inhibition [26, 60]. Healthy individuals may perceive such bimanual tasks as a single action goal, simplifying control by coupling the hands toward a common object [50]. While our findings suggest a benefit, we cannot disentangle the contribution of bimanual practice from potential benefits arising from sensory coupling via the hEXO, which warrants further investigation.

When considering patient populations, assisting one hand with the other could promote corticospinal reorganization through integration of tactile and proprioceptive feedback between the hands [27]. Similarly, others have shown in a bilateral training paradigm that using a non-paretic wrist to flex and extend a paretic wrist via a manipulandum can modulate corticomotor excitability [61]. Abdollahi et al. [62] demonstrated that interactive bilateral practice with a self-rehabilitation system could enhance error-based motor recovery in stroke survivors, while a review by van Delden et al. [63] highlighted the potential of bilateral robotic devices to promote functional gains like faster movement time and improved kinematics of the impaired limb through coupled movement strategies. Alternatively, the complementary dominance hypothesis suggests that using bilateral coordination engages the specialized roles of both hemispheres, with the left specialized for trajectory planning and the right for impedance control [19]. In patients, self-assistance could help re-engage hemisphere-specific motor functions and reinforce task-relevant control of the impaired hand.

### Transfer to unassisted unimanual performance

While these findings support the potential of self-assisted bimanual practice to engage neural mechanisms that hasten motor adaptation, it remains essential to consider whether such gains persist when self-assistance is removed, as dependency is a common concern with rehabilitation approaches that provide physical assistance [64]. We found that participants in the self-assist group maintained their gains during the unassisted trial after practice, indicating successful skill transfer rather than dependency from compensating with right-hand guidance. This suggests they were not only learning to coordinate both hands but were improving control of the artificially impaired left hand. Had performance relied solely on bimanual coordination, we would have expected a drop in accuracy or speed once assistance was withdrawn. Instead, stable unassisted performance supports the interpretation that self-assistance promoted motor adaptation and unassisted transfer of skill.

Several learning mechanisms may explain the unassisted transfer of self-assisted motor adaptation. When self-generated, assistance is likely more tightly linked to the participant’s own motor commands, which may enable faster and more accurate internal model updating [65–69]. A more efficient cerebellar-dependent updating of internal models means the effects of self-imposed external forces are readily predicted, which is important for meaningful motor adaptation during error-based learning [32]. Conversely, when a therapist or robotic system provides assistance, the sensorimotor system may struggle to predict external forces, and in turn, the uncertainty results in greater movement errors when assistance is removed as the internal model lags behind the changed dynamics [31]. Self-assistance may also have upregulated parallel model-free learning processes that support motor savings [70], driven by heightened sensorimotor engagement and agency. We also observed that the control group performed slightly slower during the last two unassisted practice blocks, suggesting a possible element of fatigue or boredom at the end of practice that was not evident in the self-assist group. Together, these mechanisms could explain both the rapid improvement and the robust unassisted transfer observed in the self-assist group.

### Neuromuscular strategies and hand aperture during self-assisted practice

To better understand how participants utilized self-assistance, we analyzed forearm muscle activity and tracked changes in hand aperture, a key feature of grasping, over the course of practice. The muscle activity data indicated that participants in the self-assist group actively used self-assistance throughout the task, roughly about 5–10% of maximum voluntary activation, with no significant changes over the course of practice. However, not all participants utilized the same neuromuscular strategy to counter the perturbation. Two participants exhibited relatively high and persistent co-contraction during self-assistance, which may represent a high impedance strategy to stiffen the hand and prevent involuntary closure from the DFES. The sustained reliance on self-assistance mirrors findings from Washabaugh and colleagues [38], who reported no reduction in muscle activity over time in individuals with stroke while training with a passive bilateral cable-driven elbow assistance device. It would be interesting to examine neuromuscular strategies further in a longer-term study to see if, over time, such participants develop less energetically expensive solutions with reduced co-activation.

The sustained use of assistance in our study may reflect the instructions, which did not direct participants to reduce assistance across trials. It remains to be seen whether participants would reduce their assistive force if explicitly instructed to assist as needed [71]. Fatigue may also have played a role; if the left hand’s extensor muscles fatigued from repeatedly opposing DFES-induced contractions, participants may have continued to assist to preserve task success. Interestingly, hand aperture did not change significantly with self-assistance or over time. Given that perceived object size does not strictly determine hand aperture [72], it is possible that participants in both groups were converging on an optimal aperture to compensate for the DFES perturbation and achieve faster, more consistent outcomes. Nevertheless, the observed improvements in performance suggest that participants adapted in other ways. Lodha et al. [73] reported that bilateral asymmetries in extension force production are more pronounced than in flexion following stroke—an asymmetry aligned with the extension demands in our DFES paradigm. Yet, they also showed that individuals post-stroke can learn to modulate forces across limbs, supporting the idea that even without large changes in EMG, our participants may have engaged in adaptive force redistribution strategies to improve grasp performance.

### Limitations

Several practical constraints and design trade-offs related to task parameters, device adaptability, acquisition of muscle activity, and psychological effects should be considered when interpreting our findings. Our sample size and practice duration were limited, so the long-term effects of self-assistance on motor learning and retention remain unknown. The reach distance was fixed rather than scaled to individual arm lengths. Although this distance was within a comfortable range for all participants and minimized the need for compensatory trunk motion, individual differences in arm length may have subtly influenced joint coordination strategies. Since the distance was consistent across all participants and conditions, we do not expect this to have introduced systematic bias. Although the hEXO was able to accommodate the hand sizes of our participants using the sliding finger cradles, it is not fully customizable to all hand morphologies. Because of large stimulation artifacts from DFES that would have made it difficult to separate the voluntary component of the muscular activity in participants’ artificially impaired arm, we only recorded muscle activity from the assisting arm. We also cannot rule out placebo effects, such as increased motivation from the perception of control over assistance through the hEXO that in turn could improve task engagement and influence motor adaptation through increased attention and task saliency, and less from assistance itself. Further study is needed to test whether the act of actively controlling assistance may enhance retention mechanisms over longer time periods or transfer benefits to different tasks beyond the skill practiced with self-assistance.

This pilot study was designed to evaluate the potential usefulness of self-assistance for rehabilitating a functional reach-to-grasp task, using a model system involving artificially impaired, right-handed healthy adults. This approach offers a controlled framework for investigating how motor adaptation unfolds during self-assistance and increases the tractability of interpreting participant responses, compared to the complex and variable physiological impairments present in real neuromuscular disorders. Still, because our model system does not fully replicate the complexity of neuromotor impairments in clinical populations, what we gain in experimental control comes at the cost of reduced generalizability. Our experiment also asked participants to complete the functional reach-to-grasp and object lift task as fast as possible, to maximize the sensorimotor challenge. Patients recovering from neurological injury typically exhibit more diverse and persistent deficits and may not be expected to perform tasks at maximal speed. Nevertheless, some evidence suggests that training with the intent to move faster can be beneficial in upper extremity rehabilitation [74].

## Conclusions

In this pilot study, conducted with a small group of healthy participants with an artificial impairment, the findings suggest that the potential benefits of self-assistance in rehabilitating reach-to-grasp actions warrant further investigation. These findings offer insights into self-assisted adaptation mechanisms, informing future studies in clinical populations with varying impairment severity and assistance needs. This work also supports the feasibility of using a hydrostatic exoskeleton (hEXO) for self-assisted rehabilitation. The hEXO’s passive design makes it suitable for home and clinical use, requiring no programming or external power. Most components are 3D-printed, making it cost-effective and addressing barriers common to traditional rehabilitation robots, such as high cost and maintenance. If further investigations, including clinical studies, provide support for a faster rate of motor adaptation with self-assistance, the approach could play a role in promoting functional independence for patients with upper extremity impairments.

## Electronic supplementary material

Below is the link to the electronic supplementary material.

Supplementary Material 1

## Data Availability

All anonymized raw data, analysis code, and data replication instructions can be found on the Open Science Framework. 10.17605/OSF.IO/3EKNM.
