# Supplementary material for: Evaluating self-assistance during functional reach with a passive hydrostatic exoskeleton under artificial impairment
Source: J Neuroeng Rehabil. 2025 Jul 16;22:163. doi: 10.1186/s12984-025-01696-8 (PMC12265121; doi:10.1186/s12984-025-01696-8)
Supplement: Supplementary file 1 — Supplementary Material 1 [file 12984_2025_1696_MOESM1_ESM.pdf]

Supplementary Material for Experiment 2

Self-Assistance During Functional Reach Using a Passive Hydrostatic Exoskeleton

Julia Manczurowsky, Henry Mayne, David Nguyen, Meghan Kenney, John Peter Whitney, and Christopher J Hasson

SECTION 1: Sensitivity Analysis for Primary Motor Adaptation Measures

Data Smoothing Prior to Exponential Fitting

We used a 20-trial moving average to filter the *ReachTime* data prior to fitting the exponentials to reduce noise and variability that might obscure the underlying adaptation trends. This approach allows the exponential fit to more accurately capture the gradual learning process while minimizing the influence of trial-to-trial fluctuations or measurement noise. To assess how smoothing affected the key measure used to test the hypothesis, the rate of improvement in reach-to-grasp time, *ReachTimeRate*, we performed a sensitivity analysis. We smoothed the data with window sizes ranging from 1 (no smoothing) to 40, redoing the exponential fitting procedure each time. The results are shown in Figure S1. As expected, the estimated adaptation rate is most sensitive to very short window sizes, but then levels off at larger windows, slightly increasing with greater window sizes. To guide our choice, we averaged the data across participants, fit a line to the adaptation rate using higher smoothing factors from 30-40, and extended that line to the lower adaptation rates. This line begins to depart from the average data at around a window size of 20. Thus, the effects of the smoothing factor are least variable above the window size of 20, with diminishing returns thereafter.

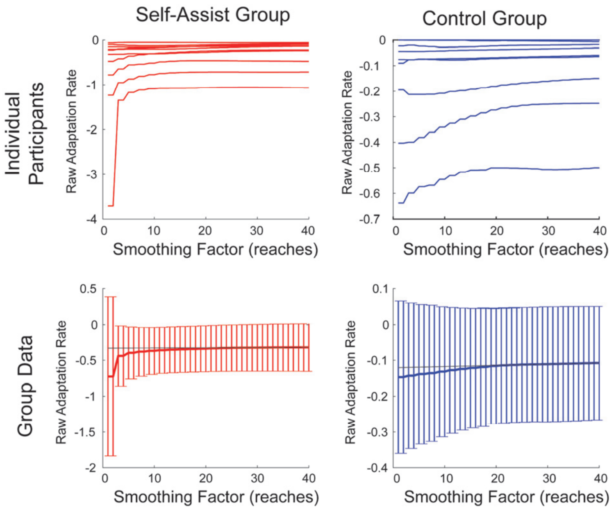

Figure S1. Effect of smoothing windows size on the adaptation rate (*ReachTimeRate*). The error bars accompanying the averaged data show the standard deviation across participants for the self-assist group (red) and control group (blue).

Effects of Different Missing Data Handling Approaches

A multiple imputation approach was used to replace missing reach times for failed reach-to-grasp attempts prior to fitting exponentials to each participant's data to derive the motor performance metrics *ReachTimeRate* and *ReachTimeAsym* (where the reach-to-grasp time levels off/asymptotes), and performing group comparisons (see main text for details). For comparison, we tried other missing data handling methods to see how different approaches affect the conclusions. This included single imputation, replacing failed trials with the maximum time-out time (2.0 s), pointwise deletion, and using regular vs. robust exponential fitting approaches. The results, detailed in Table S1, show that regardless of imputation approach and fitting type (regular or robust), the quality of the exponential fits was high (R^2 ranged from 0.68 to 0.93), and the group difference remained significant. While the pointwise deletion results were not statistically significant, the direction of the effect was consistent with the multiple imputation analysis. The discrepancy between the methods is likely due to the reduced statistical power of pointwise deletion, as it removes failed trials, which disproportionately affected one group (the self-assist group). Pointwise deletion also reduced the quality of the exponential fits.

Table S1. Comparison of results using different procedures for handling missing data.

Approach	Fit Type	*ReachTimeRate*			*ReachTimeAsym*			R^2	
		df	t	p	df	t	p	Self-Assist	Control
Pointwise Deletion	Regular	15.8	1.667	0.115	16.5	-1.506	0.151	0.52 ± 0.33	0.71 ±0.26
	Robust	15.5	1.532	0.146	17.7	-2.021	0.059	0.52 ± 0.35	0.88 ± 0.17
Max Time Imputation	Regular	14.9	3.384	*0.004	17.9	-2.297	*0.034	0.74 ± 0.30	0.63 ± 0.26
	Robust	15.3	2.965	*0.009	17.8	-2.052	0.055	0.93 ± 0.12	0.85 ± 0.27
Single Imputation	Regular	15.6	2.803	*0.013	16.4	-1.595	0.130	0.68 ± 0.28	0.72 ± 0.24
	Robust	15.5	2.720	*0.015	17.8	-2.102	*0.050	0.90 ± 0.17	0.93 ± 0.13
Multiple Imputation†	Regular	95.7	3.37	*0.001	67.5	-1.63	0.107	0.73 ± 0.23	0.72 ±0.24
	Robust	81.9	2.71	*0.008	62.9	-1.91	0.060	0.84 ± 0.25	0.94 ± 0.12

†Statistics are pooled across imputations using Rubin's Rules (see main text for details).

SECTION 2: Analysis and Results for Peak Reach-to-Grasp Speed

Although the main analysis for Experiment 2 covered multiple aspects of task performance, examining the peak speed during the reach-to-grasp action may provide further insight into motor control differences between the self-assist and control groups. Peak reach-to-grasp speed may be modulated independently of overall reach-to-grasp time.

To this end, numerical differentiation was performed on each (X, Y, Z) hand displacement component using MATLAB's gradient function, after smoothing the displacement data with a Savitzky-Golay smoothing filter (second order polynomial; window size = 7). The resulting velocity components ($\dot{X}$, $\dot{Y}$, $\dot{Z}$) were similarly smoothed and used to compute instantaneous speed as the magnitude of the velocity vector: $|v(t)| = \sqrt{(\dot{X}^2 + \dot{Y}^2 + \dot{Z}^2)}$. This yielded an estimate of movement speed that accounts for changes in all three spatial dimensions. For statistical analysis, the peak speed was computed for each successful reach-to-grasp action and averaged across each block of 20 reach-to-grasp actions. For statistical analysis a generalized linear mixed model was used with Group and Time as factors. The interaction was included and Time had repeated measures with a heterogenous autoregressive covariance structure.

Consistent with the reach-to-grasp time results (*ReachTime*), the first few reach-to-grasp attempts showed lower performance, reflected by a lower peak speed (Figure S2). The statistical results showed no effects of Group, $F(1,72) = 0.037$, $p = 0.849$, Time, $F(1,72) = 2.20$, $p = 0.095$, and no Group × Time interaction, $F(1,72) = 1.39$, $p = 0.254$.

Separate one-sample t-tests showed that, for the self-assist group, there was no difference between the last five reach-to-grasp attempts on the adaptation block (Block 4) and the first five attempts in the subsequent block when the ability to self-assist was removed (Block 5), with $t(9) = -0.643$, $p = 0.536$, demonstrating unassisted transfer. The performance of the control group was also not different between these time points, with $t(9) = -1.60$, $p = 0.145$.

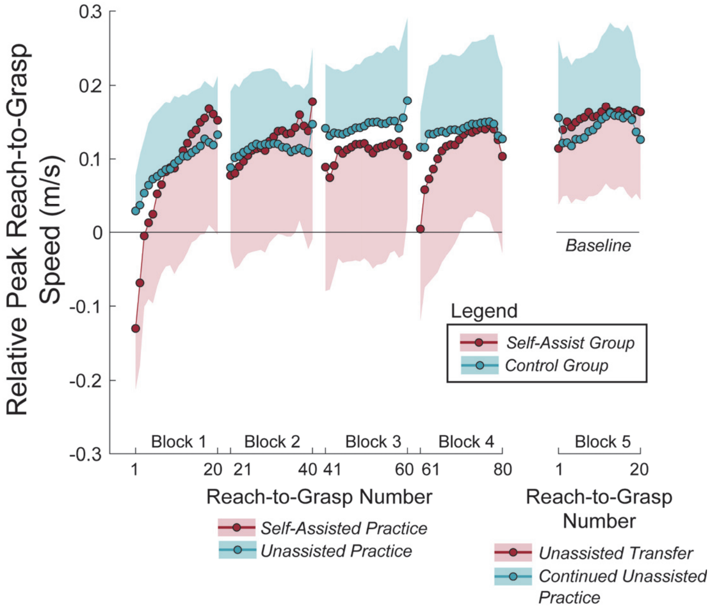

Figure S2. Relative peak reach-to-grasp speed across practice for the self-assist (red) and control (blue) groups. The line shows the mean across participants and the shading shows half of the 95% confidence interval. Only trials that had achieved a functional grasp on the object were included. Here, the averaged data were smoothed with a 10-point moving average to better visualize trends. The self-assist data does not include data from one outlier participant who had a very low (close to zero) rate of improvement in performance across the practice session.

SECTION 3: Details of Imputation-Based Inference

For each imputed dataset (i), the mean difference (Q_i) between the self-assist and control groups' *ReachTimeRate* (or *ReachTimeAsym*) and the corresponding standard error (SE_i) were computed for m imputations ($m = 100$).

The total variance (T) was computed as the sum of the average within-imputation variance and between-imputation variance, adjusted for the number of imputations:

$$T = \bar{U} + \left(1 + \frac{1}{m}\right) B$$

where $\bar{U}$ is the average of the squared standard errors from each imputed dataset (i.e., the average SE_i^2) and B is the variance of the mean differences (Q_i) across the imputed data sets.

The pooled t-statistic across imputations was calculated as $t_{pooled} = \bar{Q}/\sqrt{T}$ where $\bar{Q}$ represents the average of the group differences Q_i across the imputed datasets (the pooled mean difference).

To account for both within- and between-imputation variability when estimating statistical significance, the Barnard–Rubin adjustment was used to compute the pooled degrees of freedom. Unlike standard Rubin's Rules, which can underestimate the variability in small samples, the Barnard–Rubin method provides a more accurate degrees-of-freedom estimate by

incorporating uncertainty in the variance estimates themselves. First, the relative increase in variance (λ) due to missing data was calculated as:

$$\lambda = \frac{\left(1 + \frac{1}{m}B\right)}{T}$$

Then, the observed-data degrees of freedom (ν_{obs}) were computed as:

$$\nu_{obs} = \frac{(\nu_{com} + 1)(1 - \lambda)}{\lambda}$$

where ν_{com} is the degrees of freedom if there were no missing data. We estimated ν_{com} using the Welch–Satterthwaite approximation to account for potential inequality in group variances [54].

The final pooled degrees of freedom (ν) were calculated by combining information across imputations as follows:

$$\nu = \left(\frac{1}{\nu_{obs}} + \frac{1}{\nu_{imp}}\right)^{-1}$$

where ν_{imp} is the imputation degrees of freedom, defined as $m - 1$.

A two-tailed p-value was computed using the Student's t cumulative distribution, based on the pooled t-statistic (t_{pooled}) and the pooled degrees of freedom (ν). Effect sizes were computed for each imputed dataset using Hedges' g, which adjusts Cohen's d to correct for small-sample bias. The average Hedges' g across imputations is reported.
